# Characterization of the regulation of a plant polysaccharide utilization operon and its role in biofilm formation in *Bacillus subtilis*

**DOI:** 10.1371/journal.pone.0179761

**Published:** 2017-06-15

**Authors:** Cameron Habib, Yiyang Yu, Kevin Gozzi, Carly Ching, Moshe Shemesh, Yunrong Chai

**Affiliations:** 1 Department of Biology, Northeastern University, Boston, MA, United States of America; 2 Agricultural Research Organization The Volcani Center, Rishon LeZion, Israel; Hosei University, JAPAN

## Abstract

The soil bacterium *Bacillus subtilis* is often found in association with plants in the rhizosphere. Previously, plant polysaccharides have been shown to stimulate formation of root-associated multicellular communities, or biofilms, in this bacterium, yet the underlying mechanism is not fully understood. A five-gene *gan* operon (*ganSPQAB*) in *B*. *subtilis* has recently been shown to be involved in utilization of the plant-derived polysaccharide galactan. Despite these findings, molecular details about the regulation of the operon and the role of the operon in biofilm formation remain elusive. In this study, we performed comprehensive genetic analyses on the regulation of the *gan* operon. We show that this operon is regulated both by a LacI-like transcription repressor (GanR), which directly binds to pairs of inverted DNA repeats in the promoter region of the operon, and by the catabolite control protein A (CcpA). Derepression can be triggered by the presence of the inducer β-1,4-galactobiose, a hydrolysis product of galactan, or *in situ* when *B*. *subtilis* cells are associated with plant roots. In addition to the transcriptional regulation, the encoded ß-galactosidase GanA (by *ganA*), which hydrolyzes ß-1,4-galactobiose into galactose, is inhibited at the enzymatic level by the catalytic product galactose. Thus, the galactan utilization pathway is under complex regulation involving both positive and negative feedback mechanisms in *B*. *subtilis*. We discuss about the biological significance of such complex regulation as well as a hypothesis of biofilm induction by galactan via multiple mechanisms.

## Introduction

*Bacillus subtilis* is a Gram-positive, soil-dwelling, spore-forming bacterium, commonly found in the rhizosphere and often in association with plant roots [1, 2]. Like many other soil microorganisms, *B*. *subtilis* utilizes polysaccharides and other carbohydrate substances present in the rhizosphere as a major carbon source, many of which are from the decomposition of plant tissues [3]. Enzymes secreted by *B*. *subtilis* cells have been shown to degrade various types of plant polysaccharides and thus are of great interest in agriculture, industry, and biotechnology [4, 5]. Those secreted enzymes, and the corresponding gene clusters and pathways for plant polysaccharide utilization, have been investigated in *B*. *subtilis*, and are proposed to play important roles in helping *B*. *subtilis* cells acquire carbon sources from the environment and in promoting fitness and survival of the bacterium in the rhizosphere, though the exact mechanisms are largely unknown [3–6].

*B*. *subtilis* also serves as a model organism for biofilm studies [7–9]. Biofilms are complex multicellular communities of bacteria encased by a self-produced extracellular matrix [10, 11]. It is now believed that bacteria predominantly live as communities in the natural environments, although the exact signals and mechanisms regulating assembly of such communities are still less well understood and are a subject of ongoing investigations. While *B*. *subtilis* has long been found in the rhizosphere, only recently has evidence shown the formation of root-associated biofilms in response to signals released from plants [6, 12, 13]. Root-associated biofilms by *B*. *subtilis* function as a protective mechanism for plant hosts against pathogenic species of bacteria and fungi [13–15]. Genetic circuits that govern biofilm formation and conditions that trigger biofilm induction have been well studied in *B*. *subtilis* under laboratory conditions [8, 9, 16, 17]. On the other hand, the role of plant-released signals and the importance of plant-released nutrients on *B*. *subtilis* biofilm formation *in situ* are much less known.

**Fig 1:**
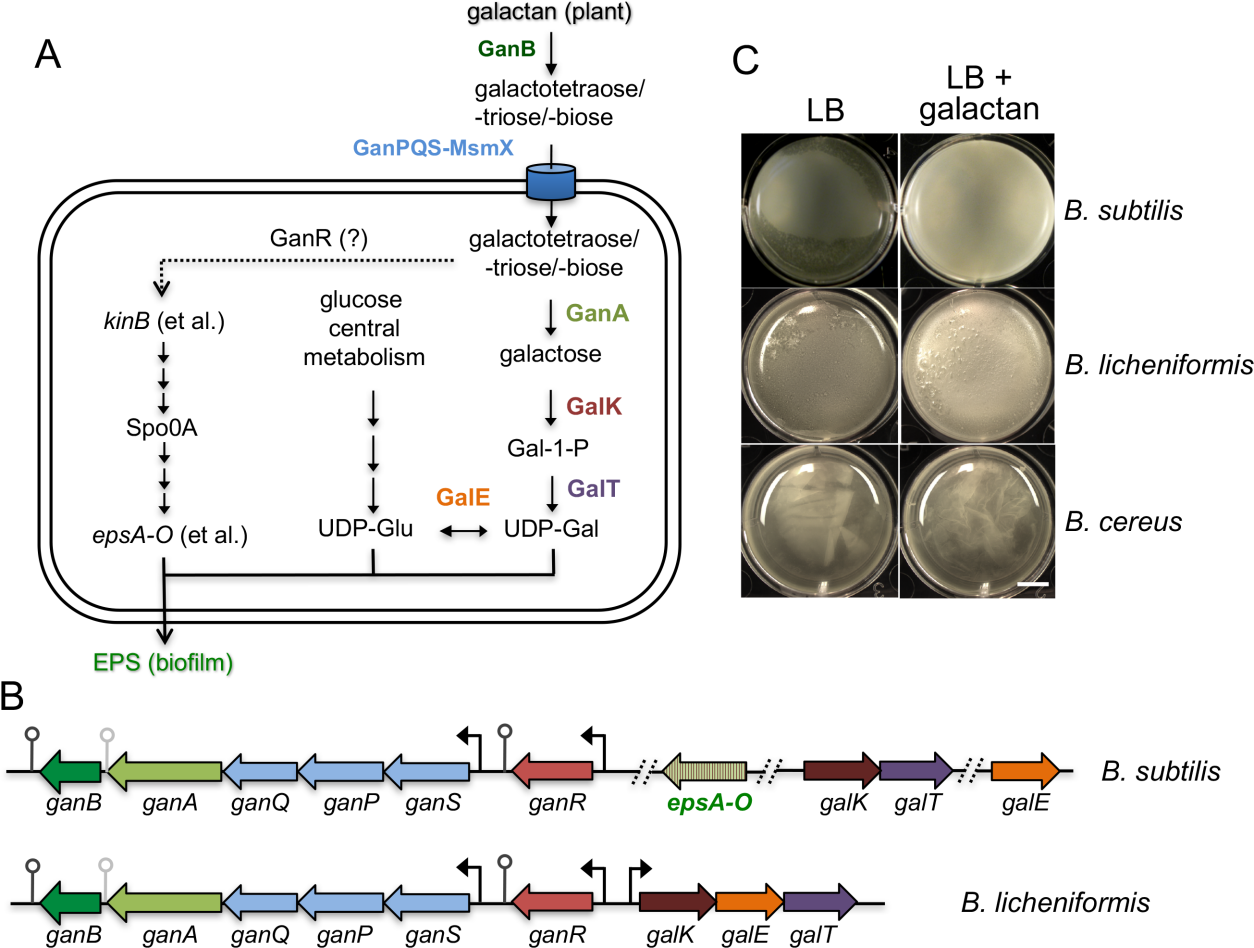
The role of the *gan* operon in galactan utilization and biofilm formation in *B*. *subtilis*. **(A)** A working model for the complete galactan utilization pathway in *B*. *subtilis*. UDP-Glu and UDP-Gal are the expected end products of galactan utilization carried out by products of the *gan* and *gal* genes. UDP-Glu and UDP-Gal are also two essential sugar nucleotide precursors for biosynthesis of exopolysaccharides (EPS) [20]. The Leloir pathway consists of *galK*, *galT*, and *galE*, whose products convert galactose to UDP-Glu [21]. EPS biosynthesis is carried out by enzymes encoded in the *espA-O* operon, which is indirectly activated by the master regulator Spo0A. Activation of Spo0A by protein phosphorylation in turn depends on multiple sensory histidine kinases including KinB [8]. It is hypothesized in this study that hydrolyzed products of galactan (e.g. galactobiose) can induce *kinB* expression via the action of GanR (see discussion). **(B)** Genetic organization of the galactan utilization genes in *B*. *subtilis* NCIB3610 and *B*. *licheniformis* ATCC8480. Putative promoters and transcription terminators are indicated. Different from *B*. *licheniformis*, in the genome of *B*. *subtilis*, the *galTK* genes and the *galE* gene are separated from the *gan* operon. In *B*. *subtilis*, the *gan* operon is also only four genes away from the *epsA-O* operon. Known or proposed functions of the *gan* and *gal* genes are as follows: *ganSPQ* encodes a permease for uptake of galactoligosaccharides; *ganA* encodes a ß-galactosidase; *ganB* encodes an endo ß-1,4-galactanse; *ganR* encodes a transcription repressor; *galK* encodes a galactokinase; *galT* for galactose-1-phosphate uridyltransferase; *galE* for the UDP-galactose-4-epimerase. **(C)** Development of pellicle biofilms by *B*. *licheniformis* ATCC8480, *B*. *subtilis* NCIB3610, and *B*. *cereus* AR156 in LB supplemented with 0.5% galactan (w/v). LB itself is a biofilm-inert medium for the above strains and used as a control. Images were taken after incubation at 30°C for 3 days. The scale bar represents 0.5 cm.

Under laboratory conditions, *B*. *subtilis* can form biofilms either as floating pellicles at the liquid-air interface, or as structurally complex surface attached colonies on solid agar media [7]. The biofilm matrix that encases individual cells within the biofilm is comprised of an amyloid-like protein fiber produced from a three-gene operon *tapA-sipW-tasA*, a hydrophobin component encoded by the *bslA* gene, and an exopolysaccharide (EPS) synthesized by products of a fifteen-gene operon *epsA-O* [8, 18, 19]. Although the structural role of the EPS in the biofilm assembly has been well established, the composition is not well characterized. In our previous study, we showed that the EPS of *B*. *subtilis* NCIB3610 is rich in glucose, galactose, and *N*-acetyl-galactose, and that specific metabolic genes responsible for making uridine-di-phosphate-galactose (UDP-Gal), one of the key sugar nucleotides for EPS biosynthesis, are essential for biofilm formation [20]. UDP-Gal is normally derived from uridine-di-phosphate-glucose (UDP-Glu) by GalE, a UDP-galactose-4-epimerase [21, 22]. UDP-Glu is in turn generated from central metabolism of glucose (Fig 1A). Alternatively, when significant amounts of free galactose or galactose-containing polysaccharides are available in the media or environments, UDP-Gal can be directly synthesized via a second route, through the conserved Leloir pathway (Fig 1A)[21]. In this pathway, galactose is first phosphorylated to galactose-1-phosphate (Gal-1-P) by the galactokinase GalK. Gal-1-P is then converted to UDP-Gal by the galactose-1-phosphate uridyltransferase GalT (Fig 1A).

While the presence of free galactose in the rhizosphere is likely limited, galactan, a polymerized form of galactose, is known to be a major component of the plant cell wall and can be readily utilized by bacteria following decomposition of plant tissues [23]. Recent studies suggest that *B*. *subtilis* cells are able to catabolize this plant polysaccharide [6, 20]. In our previous study, a presumptive five-gene *ganSPQAB* operon (hereafter the *gan* operon) was shown to be involved in utilization of plant-derived galactan and biofilm formation (Fig 1B)[6, 20]. Interestingly, this *gan* operon is only four genes away from the well-studied *epsA-O* operon (http://genolist.pasteur.fr/SubtiList/) (Fig 1B), which is involved in the EPS biosynthesis and biofilm assembly.

Genes in the *gan* operon were first studied by Errington *et al*., in which the authors showed that the *ganA* gene in the operon encodes a β-galactosidase-like enzyme, with similar activities to LacZ in *E*. *coli* [24]. Importantly, this suggests that GanA may be responsible for an endogenous baseline ß-galactosidase activity in *B*. *subtilis*. Additional studies show that *ganB* encodes a secreted endo-ß-1,4-galactanase for galactan hydrolysis, while *ganSPQ* encodes a sugar permease system for galacto-oligosaccharides [25–27]. Finally, the gene next to the *gan* operon, *ganR*, encodes a LacI-like transcription repressor shown to regulate the expression of the *gan* operon [28]. More recently, biochemical evidence suggested that the presence of galactan inhibits the direct DNA binding of the repressor GanR [29].

Despite the previous studies, a number of questions remain unaddressed, including molecular details of how exactly this operon is regulated by GanR and how regulation of the operon is coordinated with subsequent galactose metabolism and biofilm formation in *B*. *subtilis*. In this study, we further investigated those questions by performing comprehensive genetic characterizations on the regulation of the *gan* operon. Herein, we present evidence as to the role of the *gan* operon in utilization of the plant polysaccharide galactan and biofilm formation. We demonstrate that the transcriptional repressor GanR directly binds to pairs of conserved DNA motifs in the promoter regions of the *gan* operon as well as the *ganR* gene to inhibit their transcription. We also characterize the putative ligand that derepresses the operon at the transcriptional level, and a negative feedback mechanism at the protein level on GanA by the catalytic product galactose. Finally, we present a working model with integration of utilization of the plant polysaccharide galactan, cellular galactose metabolism, and biofilm assembly, during *B*. *subtilis*-plant interactions.

## Materials and methods

### Strain, media, and reagents

*Bacillus subtilis*, *B*. *licheniformis*, *B*. *cereus*, and *E*. *coli* strains were routinely cultured in lysogenic broth (LB) for normal growth. All strains used in this study are listed in Table 1. The biofilm-inducing medium LBGM is composed of LB broth (or solidified LB agar) supplemented with 1% glycerol and 100 μM MnSO_4_ [16]. If needed, antibiotics were used at the following concentrations: 5 μg ml^-1^ chloramphenicol, 0.5 μg ml^-1^ erythromycin, 10 μg ml^-1^ kanamycin, 12.5 μg ml^-1^ lincomycin, 50 μg ml^-1^ spectinomycin, and 5 μg ml^-1^ tetracycline for *B*. *subtilis* strains. For *E*. *coli* strains, ampicillin was added at 100 μg ml^-1^ and kanamycin was added at 50 μg ml^-1^. Chemicals including glucose, galactose, ß-1,4-galactobiose (CAS No. 2152-98-9), and galactan (CAS No. 38127) were purchased from Sigma-Aldrich. Restriction enzymes were purchased from New England Bio-labs (NEB, MA, USA). Oligonucleotides (S1 Table) were purchased from Integrated DNA Technologies (IDT, IA, USA). DNA sequencing was performed at Genewiz (NJ, USA).

**Table 1:** Strains used in this study.

Strains	Genotypes	References
*B*. *subtilis* PY79	A laboratory strain for genetic manipulation	[57]
*B*. *subtilis* NCIB3610	An undomesticated *B*. *subtilis* strain capable of biofilm formation	[7]
*B*. *licheniformis* ATCC8480	A biofilm-capable ATCC model strain of *B*. *licheniformis*	ATCC
*B*. *cereus* AR156	An environmental isolate of *B*. *cereus* for biological control	[58]
YC222S	An Arg^20^ to His mutation in the protein encoded by *ganR* in 3610	This study
YC453	*E*. *coli* BL21-DE3 with pET28a(P_T7_-*his6-ganR*) plasmid, Kan^R^	This study
YC1071	*amyE*::P_*ganS*_-*lacZ*::cm^R^ in 3610	This study
YC1072	*amyE*::P_*ganS*_-*lacZ*::cm^R^ in YC222S	This study
YC1073	*amyE*::P_*ganS*_-*lacZ*::cm^R^ and *ganA*::*erm*^R^ in 3610	This study
YC1074	*amyE*::P_*ganS*_-*lacZ*::cm^R^ and *ganA*::*erm*^R^ in YC222S	This study
YC1076	*ganR*^R20H^ and *amyE*::P_*spoIIA*_*-gfp*::spec^R^ in 3610	This study
YC1077	*ganA*::*erm*^R^ and *amyE*::P_*spoIIA*_*-gfp*::spec^R^ in 3610	This study
YC1078	*ganR*^R20H^, *ganA*::*erm*^R^ and *amyE*::P_*spoIIA*_*-gfp*::spec^R^ in 3610	This study
YC1085	*amyE*::P_*ganR*_-*lacZ*::cm^R^ and *ganA*::*erm*^R^ in 3610	This study
YC1086	*amyE*::P_*ganR*_-*lacZ*::cm^R^ and *ganA*::*erm*^R^ in YC222S	This study
YC1088	*amyE*::P_*ganB*_-*lacZ*::cm^R^ and *ganA*::erm^R^ in 3610	This study
YC1089	*amyE*::P_*ganB*_-*lacZ*::cm^R^ and *ganA*::erm^R^ in YC222S	This study
YC1090	*amyE*::P_*ganS*_-*lacZ*::cm^R^, *epsH*::tet^R^ in 3610	This study
YC1091	*amyE*::P_*ganS*_-*lacZ*::cm^R^, *sinR*::spec^R^, *eps H*::tet^R^ in 3610	This study
YC1092	*amyE*::P_*ganS*_-*lacZ*::cm^R^, *spo0A*::erm^R^, *epsH*::tet^R^ in 3610	This study
YC1146	*sacA*::P_*ganS*_-*lux*::cm^R^ in 3610	This study
YC1149	*amyE*::P_*yvaB*_-*lacZ*::cm^R^ and *ganA*::*erm*^R^ in 3610	This study
YC1150	*amyE*::P_*yvaB*_-*lacZ*::cm^R^ and *ganA*::*erm*^R^ in YC222S	This study
YC1151	*amyE*::P_*yvaB*_-*lacZ*::cm^R^, *ganA*::*erm*^R^, *ykvE*::*tet*^R^ in 3610	This study
YC1248	*amyE*::P_*ganS*_-*lacZ*::cm^R^, *degU*::tet^R^ in 3610	This study
YC1249	*amyE*::P_*ganS*_-*lacZ*::cm^R^, *ccpA*::erm^R^, *epsH*::tet^R^ in 3610	This study
YCN217	*ganR*::*erm*^R^ in 3610	This study
CH092	*amyE*::P_*ganS*_*mut1*-*lacZ*::cm^R^ and *ganA*::*erm*^R^ in 3610	This study
CH093	*amyE*::P_*ganS*_*mut2*-*lacZ*::cm^R^ and *ganA*::*erm*^R^ in 3610	This study
CH094	*amyE*::P_*ganS*_*mut3*-*lacZ*::cm^R^ and *ganA*::*erm*^R^ in 3610	This study

### Pellicle development

Cells were inoculated from colonies on an overnight LB agar plate into 3 mL of LB broth and grown with shaking at 37°C to log phase. Cells were then subcultured 1:1000 into 7 mL of LB in a 6-well polyvinyl plate (VWR, PA, USA), with and without 0.5% galactan (w/v). Plates were incubated in static conditions at 30°C for 3 days and were then imaged using a Sony NEX-7 camera.

### Bioinformatics analysis

A bioinformatics search for potential GanR binding sequences in the *B*. *subtilis* genome was performed using the consensus DNA motif (AGTAAA-N4-TTTACT) identified in this study on the Subtilist Server website (http://genolist.pasteur.fr/SubtiList/) with the pattern search function. The criteria for the pattern search was set so that one nucleotide mismatch was allowed in each inverted repeat and the putative motifs should be located in the intergenic region within 300-bp distance upstream from the start codon of the putative open reading frame. Motif patterns were confirmed using the recursive prokaryotic sampler mode of Gibbs Motif Sampler on the identified motif-bearing promoters [30, 31]. Predictions of the secondary structure and hairpin formation were performed using RNAstructure (http://rna.urmc.rochester.edu/RNAstructureWeb/) by applying the standard analysis parameters [32].

### Strain construction

General methods for molecular cloning followed published protocols [33]. SPP1 phage-mediated transduction was used to transfer antibiotic-marked DNA fragments among different strains [34, 35]. Long-flanking PCR mutagenesis was used to generate insertional deletion mutations [36].

YC222S is a *B*. *subtilis* 3610 derivative containing a single nucleotide change in the coding sequence of *ganR*, which results in Arg^20^>His change in the amino acid sequence of GanR. This strain was initially obtained in a random screen of *B*. *subtilis* 3610 cells on LB+X-gal (40 μg ml^-1^) plates for blue colonies as an indication of enhanced production of endogenous β-galactosidase activities (e.g. from GanA whose gene is repressed by GanR). The mutation in YC222S resulting in blue colonies on LB+X-gal plates was later mapped to a single nucleotide change in the *ganR* gene, which results in Arg^20^>His change in the amino acid sequence of GanR. The general procedures for mapping the random mutation followed the protocol published previously [37]. To construct a deletion mutation in the *ganR* gene in the *B*. *subtilis* 3610 background, the deletion strain of *ganR* (Δ*ganR*::*erm*^R^) in the *B*. *subtilis* 168 background was obtained from Bacillus Genetic Stock Center (BGSC). The mutation cassette (Δ*ganR*::*erm*^R^) was then introduced into 3610 by SPP1 phage mediated transduction.

To construct the *lacZ* fusion reporter for the *gan* operon (P_*ganS*_-*lacZ*), the promoter of the *ganS* gene was PCR amplified by using 3610 genomic DNA as the template and the primers P_*ganS*_-F1 and P_*ganS*_-R1. The PCR products were purified using gel purification kit (Qiagen). Purified PCR products and pDG268 plasmid [38] were digested with EcoRI and BamHI, gel-purified, and ligated by T4 DNA Ligase. Ligation was transformed into competent *E*. *coli* DH5α cells by following the published protocol [33]. Transformants were selected for on LB agar plates containing 100 μg ml^-1^ ampicillin. Colonies were selected and cultured in 3 mL LB with 100 μg ml^-1^ ampicillin overnight at 37°C and recombinant plasmids were purified by Miniprep Plasmid Purification kit (Qiagen). Recombinant plasmids were verified by gel electrophoresis after restriction digestion and DNA sequencing. The recombinant plasmid was then introduced into *B*. *subtilis* strain PY79 by genetic transformation by following a published protocol [39]. Integration of the transcriptional reporter fusion at the *amyE* locus (encoding a starch-degrading amylase) on the PY79 chromosome was verified on LB plus starch plate for loss of amylase activities. The reporter fusion was then introduced into 3610 and its derivatives by SPP1-phage transduction [35]. Construction of the *lacZ* fusion reporters for the *ganR*, *ganB*, and *yvaB* genes (creating P_*ganS*_-*lacZ*, P_*ganB*_*-lacZ*, and P_*yvaB*_*-lacZ*, respectively) followed very similar procedures except that different primers (S1 Table) were used accordingly during PCR amplification.

To construct the luciferase reporter for the *gan* operon (P_*ganS*_-*lux*), the *ganS* promoter was PCR amplified by using 3610 genomic DNA as the template and the primers P_*ganS*_-F1 and P_*ganS*_-R2. The resulting PCR products were digested with EcoRI and NotI and cloned into the reporter plasmid pAH328, which bears a promoter-less *lux* reporter [40]. The rest of the procedures for introducing the reporter fusion into *B*. *subtilis* was similar to what was described above except that the chromosomal integration of the reporter fusion was at the *sacA* locus in *B*. *subtilis*.

### Site-directed mutagenesis

Construction of point mutations was completed by site-directed mutagenesis utilizing a modified protocol published by Ho *et al*. [41]. Primers were designed to alter two of the six bases in three separate binding domains within the P_*ganS*_ in two fragments, overlapping at the modification site by use of P_*ganS*_-F1 and corresponding P_*ganS*_-M reverse (R) primer for upstream fragment, and P_*ganS*_-R1 and corresponding P_*ganS*_-M forward (F) primer for downstream fragment (S1 Table). The initial PCR amplification of P_*ganS*_ was performed from 3610 genomic DNA template using OneTaq (NEB) and subsequently purified by gel electrophoresis. To join upstream and downstream DNA fragments for a full-length product, DNA products obtained in the initial PCR were added as templates to a subsequent PCR round, without the addition of primers. Resulting full-length mutagenic P_*ganS*_ DNA products were purified using gel electrophoresis and restriction digested with EcoRI and BamHI, and ligated into pDG268 with T4 DNA Ligase. Ligated plasmid was transformed into *E*. *coli* strain DH5α and the recombinant plasmid was purified by Miniprep Kit (Qiagen). Resulting purified plasmid was verified via DNA sequencing and transformed into *B*. *subtilis* strain PY79 and moved by SPP1-phage mediated transduction into *B*. *subtilis* strain 3610 using published protocols [35, 39].

### Assays of β-galactosidase activities

Assays were conducted as previously described [42]. Cells were cultured in LB or LBGM medium at 37°C in a water bath with shaking. One milliliter of culture was collected at each indicated time point and cells were centrifuged down at 5000 rpm for 10 min. Cell pellets were suspended in 1 ml Z buffer (40 mM NaH_2_PO_4_, 60 mM Na_2_HPO_4_, 1 mM MgSO_4_, 10 mM KCl, and 38 mM ß-mercaptoethanol) supplemented with 200 μg ml^-1^ lysozyme. Resuspensions were incubated at 37°C for 15 min. Reactions were started by adding 200 μL of 4 mg ml^-1^ ONPG (2-nitrophenyl-ß-D-galactopyranoside) and stopped by adding 500 μL of 1 M Na_2_CO_3_. Samples were briefly centrifuged down at 5000 rpm for 1 min. The soluble fractions were transferred to cuvettes (VWR), and absorbance of the samples at 420 nm was recorded using a Bio-Rad Spectrophotometer. The ß-galactosidase specific activity was calculated according to the equation (Abs_420_ / time x OD_600_) x dilution factor x1000. Assays were conducted at least in triplicate.

### Assays of galactose inhibition on GanA

YC222S (the *ganR* ^R20>H^ point mutant for overexpression of endogenous ß-galactosidase GanA) or YC1074 (the double mutant of *ganR*^R20>H^ and Δ*ganA* bearing P_*ganS*_-*lacZ* for overexpression of *E*. *coli* β-galactosidase LacZ) were used to compare feedback inhibition of the catalytic product galactose to either GanA of *B*. *subtilis* or LacZ of *E*. *coli*. Cells were grown in LB and harvested at OD600 = 1. Cell pellets were similarly suspended in Z buffer supplemented with 200 μg ml^-1^ lysozyme. Resuspension was incubated on ice for 30 min and treated with sonication to completely lyse the cells. Resuspension was centrifuged down at 4°C for 10 min and the supernatant was transferred to clean tubes for the assays.

Resuspension was distributed in 1 mL aliquot into each test tube. In each test tube, varying concentrations of galactose (from 2.5 to 20 mM) were added. ONPG (2-nitrophenyl-ß-D-galactopyranoside) was then added to the mixture at a final concentration of 2.5 mM to start the reaction. After about 2 min of incubation, the reaction was stopped by adding 500 μL of 1 M Na_2_CO_3_. The ß-galactosidase specific activity of the samples was measured similarly as described above. Assays were conducted at least in triplicate.

### Protein purification

The *E*. *coli* strain YC453 was used for the production of His_6_-GanR fusion proteins. 500-mL cultures were grown in LB broth supplemented with 50 μg ml^-1^ kanamycin at 30°C to an OD_600_ of 0.5. IPTG was then added to a final concentration of 1 mM and cultures were incubated at 30°C for two more hours. Cells were harvested and washed once with 50 mL cold phosphate buffer (20 mM sodium phosphate, 200 mM NaCl, 10% glycerol, 1 mM PMSF, pH 7.4). Cell pellets were suspended in 5 mL of cold phosphate buffer supplemented with 200 μg ml^-1^ of lysozyme and incubated on ice for 30 min. Lysed cells were further disrupted on ice using sonication. Cell lysates were centrifuged at 5000 rpm for 5 min to remove cell debris and were further ultracentrifuged at 35,000 rpm for 30 min at 4°C. Soluble fractions were transferred to clean cold tubes.

One mL of Ni-NTA agarose beads (Qiagen) was added to the cleared lysate and samples were gently rotated for 2 h at 4°C. The lysate/bead mixture was then loaded onto a column and washed five times, each time with two bed volumes of wash buffer (20 mM sodium phosphate, 300 mM NaCl, 10% glycerol, 20 mM imidazole, pH 8.5). The column was eluted with 5 bed volumes of elution buffer (20 mM sodium phosphate, 300 mM NaCl, 10% glycerol, 300 mM imidazole, pH 8.5). Collected fractions were run on a 12% SDS-PAGE to examine the protein purification. Fractions containing the affinity-purified proteins were pooled and dialysed against a dialysis buffer (20 mM sodium phosphate, 300 mM NaCl, 0.3 mM DTT, 10% glycerol, pH 7.4) overnight. The final protein preparation was quantified using a BCA Protein Assay Kit (Pierce, IL, USA). Proteins were stored in 25% glycerol at -80°C.

### Electrophoretic mobility shift assays (EMSA)

For assays of GanR binding to the promoters of the *gan* operon (P_*ganS*_), the *ganR* (P_*ganR*_), and *yvaB* (P_*yvaB*_) genes, DNA probes used in the assays were generated by PCR using 3610 chromosomal DNA as the templates, and using primers P_*ganS*_-F1 and P_*ganS*_-R1 (for P_*ganS*_), P_*ganR*_-F1 and P_*ganR*_-R1 (for P_*ganR*_), and P_*yvaB*_-F1 and P_*yvaB*_-R1 (for P_*yvaB*_). Each PCR product was gel purified, resuspended in ddH_2_O, and the concentration was quantified using Nanodrop (Fisher Thermo Scientific). DNA protein binding reactions were incubated in 10 μL of binding buffer (10 mM Tris•HCl, 50 mM NaCl, 1 mM EDTA, 5% glycerol, 1 mM DTT, 10 μg ml^-1^ BSA). Various concentrations of His_6_-GanR proteins (from 1, 3, 10, to 30 μM) were added to approximately 1 μg (approximately 0.3 μM) DNA probe and incubated on ice for 20 min. Reaction mixture was size-fractionated on a 6% polyacrylamide gel (in 1X TBE buffer) at 250 V. The gel was post-incubated with distilled water supplemented with ethidium bromide (EB) for 20 min and then wash-incubated with distilled water without EB for another 10 min. Image of the gel was taken using GelDoc-It and captured by the VisionWork software (UVP, USA).

For EMSA performed using fluorescent DNA probes, the fluorescent probes were generated by PCR amplification from appropriate plasmid templates (WT, Mut1, and Mut2 in this study) using a 5’ Cy3 labeled primer (Integrated DNA Technologies, USA) and purified using the gel purification kit (Qiagen). Resulting DNA was quantified by Nanodrop and equilibrated to 50 ng μL^-1^. Approximately 16 ng of DNA was incubated with a gradient of GanR proteins (from 0.08 to 4 μM). To reduce non-specific binding, poly-dIdC was added to all samples. Reaction mixture was size-fractionated on a 6% polyacrylamide gel (in 0.5X TBE buffer) at 100 V for 2 h at 4°C. The resulting gel was imaged using ChemiDoc MP (Bio-Rad, USA), and quantified by Image Lab software V6.0 (Bio-Rad, USA).

### Luciferase assays

Reporter strains were grown overnight in shaking at 25°C in 3 mL of LB. Cultures were equalized for OD_600_ and sub-cultured into 3 mL of LB and allowed to grow for 4 h in shaking at 37°C to an OD_600_ of approximately 0.7. Equalized cultures were subcultured 1:100 in triplicate into 175 μL of the minimal defined MSgg medium [7] in a 96-well tissue culture plate (VWR). Test reagent was added in 5 μL volume to indicated concentration. Bioluminescence was read every 30 min with an integration time of 5 sec under a 135 gain and 4.5 mm read height in a BioTek Synergy H1M plate reader shaking constantly at 37°C.

### Preparation of tomato root exacts

Tomato growth condition was modified from Chen *et al*. [12]. Briefly, tomato seeds (Lycopersicon esculentum Miller) were surface sterilized in sodium hypochloride (10% active chlorine) for 10 min and followed by five subsequent washing steps with sterile water. Sterilized seeds were then transferred onto 0.7% Murashige and Skoog (MS) agar plates [43] for germination and incubated at 25°C for 3~4 days until the length of tomato roots reached about 3 cm. The seedlings were transplanted into 12-well plates containing 4 mL of MS medium in each well, and incubated at 25°C in a shaker at 60 rpm with photoperiod of 16 h of light and 8 h of dark for two days. To prepare homogenized plant root exacts for test of the *gan* operon induction, 10 plant roots were collected and washed three times in sterile phosphate buffered solution (PBS). Washed roots were placed into a glass tissue grinder with 5 mL of sterile PBS and homogenized manually. The resulting solution was filter sterilized twice through a 0.2 μm filter and stored at 4°C until use.

### Bacteria root hybridization

Equalized OD_600_ cultures were prepared as described above and subcultured 1:100 into 7 mL of MS medium in 6-well tissue culture plates as described by Chen *et al*. [12]. Plants were grown according to the protocol described in the preparation of tomato root exact above and each placed into a separate inoculated well in triplicate. Sterilized bamboo applicator sticks (Fisher Scientific, MA, USA) were used as a control of abiotic surface and sectioned into 2 cm fragments and two were placed into an inoculated well as control substance in triplicate. Plates were incubated at 25°C in a shaker at 60 rpm with photoperiod of 16 h of light and 8 h of dark for two days. Plant root and control substance were gently rinsed twice in sterile PBS, placed into a 2 mL microcentrifuge tube with 1.25 mL sterile PBS, and vortexed for 30 sec. Tubes were spun down in a centrifuge for 1 min at 16,000 rpm and washed 3 times in sterile PBS. Resulting cultures were collected and processed according to protocols described above in assays of ß-galactosidase activities.

## Results

### Addition of the plant polysaccharide galactan stimulates biofilm formation in both *B*. *subtilis* and *B*. *licheniformis*

In our previous study [20], we presented evidence that the *gan* operon is part of the pathway involved in utilization of plant polysaccharide galactan; growth of the *B*. *subtilis* mutant (Δ*ganSPQAB)* was partially impaired when cells were grown in a minimal medium with galactan as the sole carbon source [20]. In this study, we further found that addition of galactan (0.5%, w/v) in LB, a less favorable medium for biofilm formation, promoted pellicle biofilm formation in *B*. *subtilis* NCIB3610 (hereafter 3610). In addition, we also tested whether galactan can stimulate pellicle biofilm formation in *B*. *licheniformis* ATCC8480, which contains a highly conserved *ganSPQAB* operon in its genome (Fig 1B), and in *B*. *cereus* AR156 whose genome does not have a homologous *gan* operon (personal observation). As shown in Fig 1C, in *B*. *licheniformis*, galactan promoted formation of floating pellicles that were thick in biomass and tightly attached to the edge of the wells, whereas in *B*. *cereus*, very little floating pellicles were seen. Note that addition of galactan did not alter the growth rate of the above bacteria in LB (data not shown). Our results suggest that the conserved *gan* operon may play a role in biofilm formation in *Bacillus* species.

### GanR acts as a transcriptional repressor for the *gan* operon and the *ganR* gene

**Fig 2:**
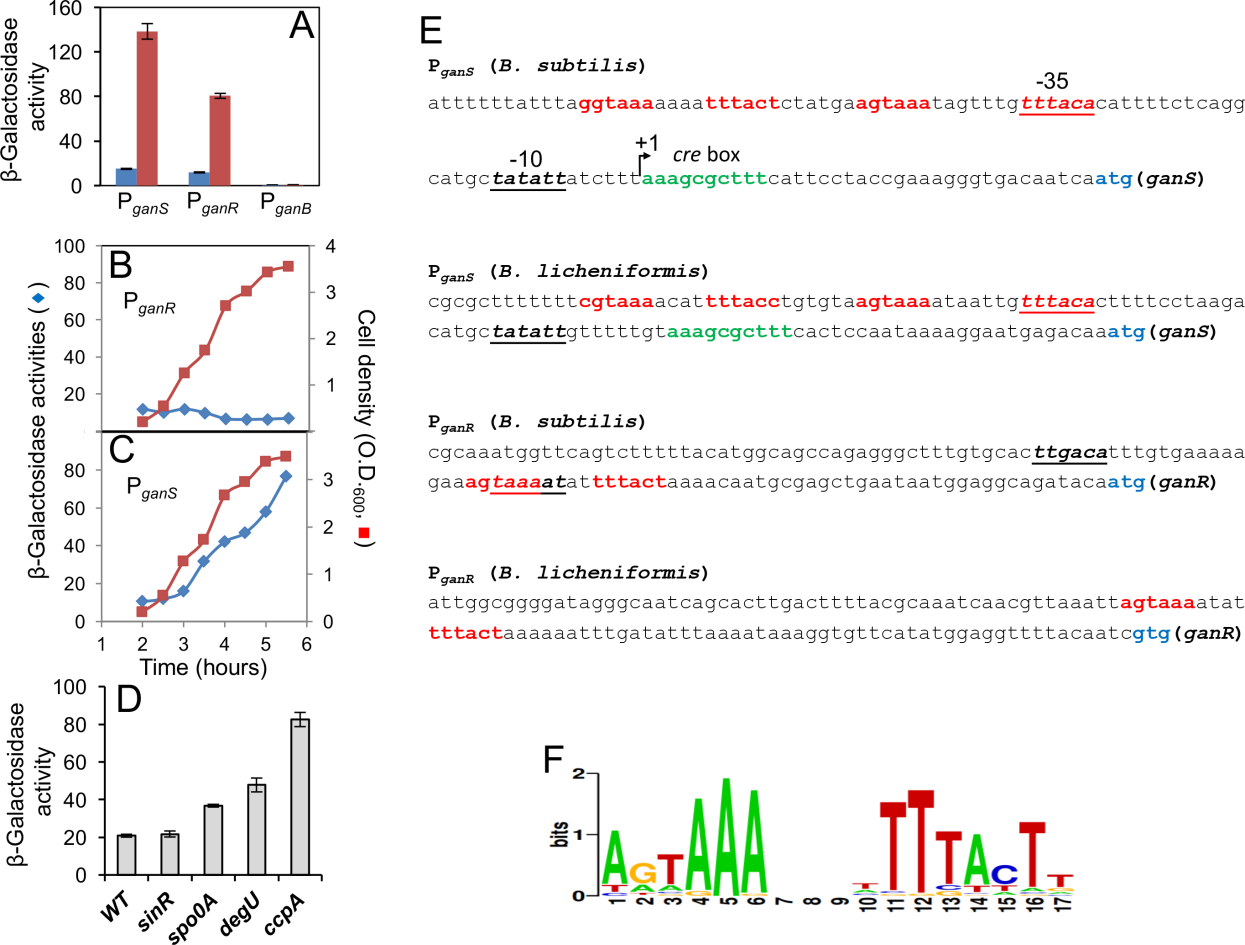
GanR represses the *gan* operon and the *ganR* gene. **(A)** Assays of β-galactosidase activities by the reporter strains bearing either P_*ganS*_-*lacZ*, or P_*ganR*_-*lacZ*, or P_*ganB*_-*lacZ* in the wild type strain (blue bars; YC1073, YC1085, and YC1088) and the *ganR* mutant (red bars; YC1074, YC1086, and YC1089). A deletion mutation in *ganA* was also introduced into the above strains. Cells were grown in LB shaking broth to OD_600_ = 1 before harvest and analyses. Assays were done in triplicates and error bars represent standard deviations. **(B-C)** Assays of ß-galactosidase activities by the wild type reporter strains bearing either P_*ganS*_-*lacZ*(YC1073, panel B) or P_*ganR*_-*lacZ*(YC1085, panel C). Cells were grown in LB shaking culture over a period of 5.5 hours after inoculation. Both culture densities (red squares, right-hand y-axis) and ß-galactosidase activities of cells (blue diamonds, left-hand y-axis) were measured. Assays were repeated multiple times and representative data was shown here. **(D)** Assays of ß-galactosidase activities by the P_*ganS*_-*lacZ* reporter strains in the wild type background (YC1071), the Δ*sinR* (YC1091), Δ*spo0A* (YC1092), Δ*degU* (YC1248), and Δ*ccpA* (YC1249) mutants. The *ganA* deletion mutation was not introduced into the above strains. In some mutants, an *epsH* deletion mutation was also introduced to prevent cell aggregation during shaking growth [45]. Cells were grown in LB shaking culture to OD_600_ = 1 before harvest and analyses. Error bars represent standard deviations. **(E)** Display of the promoter regions of *ganS* and *ganR* from *B*. *subtilis* NCIB3610 and *B*. *licheniformis* ATCC8480. The inverted repeats are highlighted in red, the -35 and -10 motifs of the sigma A-dependent promoter are underlined and shown in italic. ATG or GTG start codons of *ganS* or *ganR* are highlighted in blue. The *cre* box for putative CcpA binding sequences in the *ganS* promoter regions is highlighted in green. The transcriptional start of the *ganS* gene in *B*. *subtilis* was determined in a very recent study [29] and labeled as +1. **(F)** The consensus DNA motif logo was generated from a multiple sequence alignment of the putative motifs from the selected promoters using WebLogo [31]. The height of each stack, displayed in bits, is representative of the frequency of the nucleotide in the motif.

Because of the importance of the *gan* operon in plant polysaccharide utilization and biofilm formation, we decided to further characterize the regulation of this operon. Evidence from several previous studies suggested that GanR, a LacI-family transcription repressor, whose gene lies next to the *gan* operon (Fig 1B), negatively regulates the operon [28, 29]. We sought to confirm this regulatory effect of GanR, and in addition to test the regulation of the *ganR* gene itself. Two reporter fusions, P_*ganS*_*-lacZ* and P_*ganR*_*-lacZ*, were constructed and introduced into both the wild type strain and the *ganR* mutant of *B*. *subtilis* 3610, respectively. For the *ganR* loss-of-function mutant, we obtained two different isogenic strains. For one (YCN217), an insertional deletion mutation in *ganR* was created by replacing the coding sequence of *ganR* with an erythromycin resistance marker (∆*ganR*::*erm*^R^). For the other, a point mutant of *ganR* (Arg^20^>His) was obtained separately from a genetic screen for enhanced endogenous β-galactosidase activities in *B*. *subtilis* isolates (see Materials and Methods). The spontaneous mutation that caused greatly enhanced endogenous β-galactosidase activities in *B*. *subtilis* was later mapped to a single nucleotide change in the *ganR* coding sequence. As the result of the single nucleotide change, an arginine residue that is highly conserved in the putative DNA binding domains of this family of LacI-like transcription repressors in *B*. *subtilis* is changed to histidine (Arg^20^>His)(S1 Fig). This change likely abolishes the ability of GanR on repression. Indeed the point mutant of *ganR* and the deletion mutant showed virtually identical activities of the *gan* operon (S2A Fig). Due to the benefit of using a marker-less mutation in the sequential construction of strains bearing multiple drug-cassette marked mutations onto the chromosome, we decided to use the point mutant of *ganR* (Arg^20^>His) in this study in most applications where *ganR* loss of function mutation was needed. Also note that in this study, unless indicated differently, a *ganA* insertion deletion (*ganA*::*erm*^R^) was introduced into all *lacZ* reporter strains in order to eliminate any endogenous β-galactosidase activities [28]. We next compared the activities of *ganS* and *ganR* in the wild type strain and the *ganR* mutant by assaying β-galactosidase activities of the above reporter strains. Our results confirmed a strong negative regulation of the *gan* operon by GanR since the activity of P_*ganS*_-*lacZ* increased more than 5-fold in the *ganR* mutant (red bar, Fig 2A) compared to that in the wild type strain (blue bar, Fig 2A). GanR also seems to negatively regulate its own gene since the activity of P_*ganR*_-*lacZ* was at least 3-fold higher in the *ganR* mutant (red bar, Fig 2A) than in the wild type (blue bar, Fig 2A). Thus, *ganR* is under a self-negative feedback regulation.

Bioinformatics analysis of the *gan* operon shows a 79-bp intergenic region between *ganB* and *ganA*, present in *both B*. *subtilis* and *B*. *licheniformis* genomes (S3A and S3B Fig), and in other *Bacillus* species (data not shown). This leads to the question whether the operon is driven from a single promoter, P_*ganS*_, or in addition to that, an internal promoter could exist within the operon and drive expression of just the downstream *ganB* gene. Such an internal promoter would provide additional regulation to the operon. To test this possibility, we constructed a similar *lacZ* reporter fusion (designated as P_*ganB*_*-lacZ*) by amplifying a 180-bp DNA sequence covering the intergenic region between *ganA* and *ganB* and the 3’ end of the *ganA* coding sequence by PCR, fusing it to *lacZ*, and introducing this fusion into the wild type strain and the *ganR* mutant, respectively. However, in the ß-galactosidase assays, only background activities were detected in the P_*ganB*_-*lacZ* reporter strains in both the wild type and the *ganR* mutant background (Fig 2A). This result argues against the presence of an internal promoter in the intergenic region of *ganA* and *ganB*.

Although no internal promoter activity was detected, we noticed that the intergenic region contains a putative Rho factor-independent transcription terminator immediately downstream of *ganA* (S3A and S3C Fig). This points to the possibility of a transcriptional attenuation mechanism within the presumptive *gan* operon. A similar terminator/attenuator-like structure can also be found in the intergenic region of *ganA* and *ganB* in *B*. *licheniformis* (S3B Fig). A recent study investigated global gene expression in the *B*. *subtilis* strain 168 under a large variety of different media conditions by using tiling microarray [44]. The publically available original raw transcription data from that study showed transcription attenuation immediately downstream of *ganA* under all 8 tested conditions [44]. This may provide evidence for the existence of a transcription attenuation mechanism in the *gan* operon.

### The *gan* operon is also under the control of catabolite repression

We carried out similar assays to determine the expression profile of *ganS* and *ganR* over a period of time during shaking growth by using the two reporter strains (P_*ganS*_-*lacZ* and P_*ganR*_-*lacZ*) in the wild type background. Interestingly, the observed expression profile of *ganR* was quite different from that of *ganS*; *ganR* was expressed at a relatively low and constant level (diamonds in blue, Fig 2B) while the expression of *ganS* increased several folds over time (diamonds in blue, Fig 2C). This may suggest that self-regulation of *ganR* (by GanR) quickly achieves equilibrium. Hence *ganR* expression remains relatively constant. While for *ganS*, there is likely another regulation in addition to GanR repression, causing increased activities of P_*ganS*_*-lacZ* over time. We subsequently tested possible regulation of the *gan* operon by several master regulators known to function during growth transition and have roles in biofilm formation in *B*. *subtilis*, namely SinR, Spo0A, DegU, and CcpA [45–48]. The P_*ganS*_-*lacZ* reporter fusion was introduced into each of the deletion mutants for the corresponding master regulator and the activities of P_*ganS*_-*lacZ* in those mutants (the *ganA* gene is intact in those strains) was compared. While both Spo0A and DegU seemed to have a mild effect on *ganS*, a significant regulation was seen by CcpA, the carbon catabolite repressor (Fig 2D). Previous genome wide studies identified a putative high affinity *cre* box within the *ganS* promoter region, with a strong repression observed by microarray upon CcpA induction [49]. Our finding here confirmed the presence of this box and regulation of CcpA on *ganS* (*cre* box, Fig 2E), with the motif found to be overlapping with the recently experimentally confirmed transcription start site of *ganS* [29] (+1, Fig 2E). This indicates that the *gan* operon is regulated by both GanR and catabolite repression (CcpA). In contrast, *ganR* was not found to be regulated by CcpA nor can a *cre* box be identified in the *ganR* promoter (data not shown).

### GanR represses by direct DNA binding

A bioinformatics analysis of the promoter regions of *ganS* and *ganR* in *B*. *subtilis* was performed to identify any putative DNA motifs, which could support direct GanR binding. Indeed, a consensus DNA motif “AGTAAA-(4-7nt)-TTTACT” (Fig 2F) with two inverted repeats was found to be present twice immediately upstream of the -35 motif of the *ganS* promoter and once within the *ganR* promoter (Fig 2E). Similar arrangements of these DNA motifs were also found in the *ganS* and *ganR* homologous genes in *B*. *licheniformis* (Fig 2E) and other closely related *Bacillus* species (data not shown), despite a significant difference in overall DNA sequences between these species. Given the location of these DNA motifs relative to the transcriptional start site of the genes, binding of GanR would block the entry of the RNA polymerase, resulting in transcriptional repression as seen.

**Fig 3:**
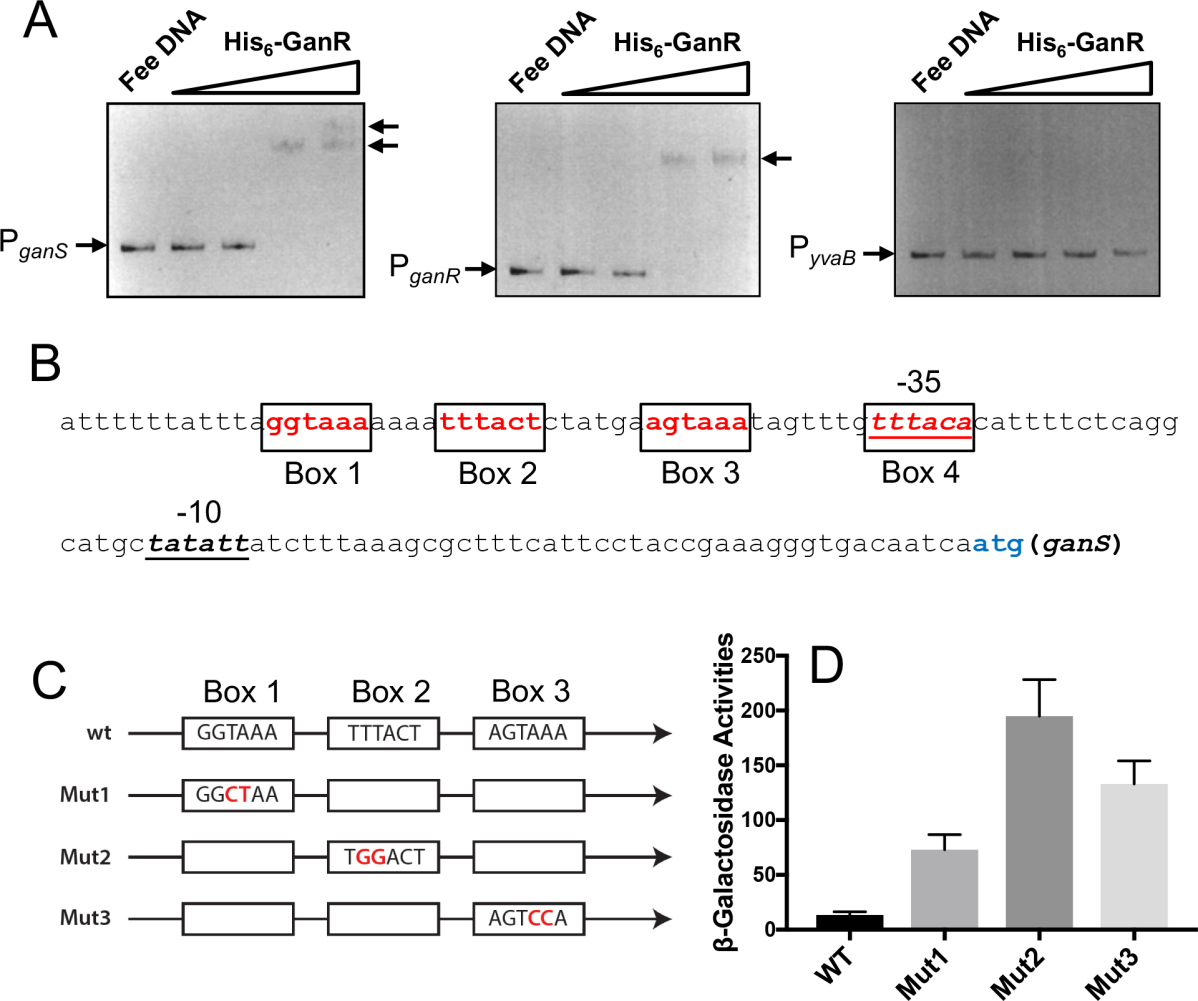
GanR directly binds to the promoters of *ganS* and *ganR*. **(A)** Gel mobility shift assays to probe binding of purified His_6_-GanR to the DNAs containing the promoter sequence of *ganS*, *ganR*, or *yvaB*. Approximately 1 μg (approximately 0.3 μM) of DNA was added to each lane, His_6_-GanR was added at increasing concentrations from 1, 3, 10, to 30 μM, no protein was added in the control lanes (left-most). Mobility retarded DNA bands were indicated by arrows. **(B)** The DNA sequence of the *ganS* promoter in *B*. *subtilis*. The -35 and -10 motifs of the sigma A-dependent promoter are highlighted in italic. The putative GanR binding motifs are labeled from Box1 to Box4. **(C)** A schematic display of site-directed mutagenesis on the putative GanR binding sites in the *ganS* promoter (mut1, mut2, and mut3). Letters in red are the introduced nucleotide changes in each of the boxes. Mutagenesis in Box4 was avoided due to overlap with the -35 motif of the promoter. **(D)** Assays of ß-galactosidase activities by the P_*ganS*_-*lacZ* reporter strains with either the wild type promoter sequence of *ganS*, or with various sited-directed mutations shown in (C). Cells were grown in LB shaking culture to OD_600_ = 1 before harvest and analyses. Error bars represent standard deviations from four independent analyses.

It seems plausible to assume that GanR represses transcription of the *gan* operon and the *ganR* gene through direct binding to those DNA motifs in the promoters (Fig 2E). We decided to test this by performing Electrophoretic Mobility Shift Assays (EMSA). The promoter regions of *ganS*, *ganR*, and *yvaB* (used as a control) were PCR amplified by using appropriate primers (S1 Table) and 3610 genomic DNA as the template. His-tagged GanR proteins were expressed from a recombinant *E*. *coli* strain and affinity-purified (S4A Fig). DNA probes were incubated with a gradient of his-tagged GanR proteins in the EMSA. With increasing amounts of GanR added, shift of both P_*ganS*_ and P_*ganR*_ DNA probes was observed, indicating a direct binding of GanR to both promoters (Fig 3A). Furthermore, in the case of P_*ganS*_, two distinct shifted DNA bands were observed when the highest amount of GanR was added (indicated by arrows, left-hand panel, Fig 3A), suggesting two binding events by GanR, while for P_*ganR*_, only one shifted DNA band was observed when the same conditions applied (indicated by the arrow, middle panel, Fig 3A). These molecular details are consistent with our bioinformatics analysis, showing that the *ganS* promoter contains two pairs of the inverted repeats while the *ganR* promoter has only one (Fig 2E), which were not revealed in any of the previous studies.

In addition, the promoter of the *yvaB* gene (P_*yvaB*_) was used as a negative control in the EMSA (Fig 3A). Interestingly, P_*yvaB*_ also contains very similar inverted repeats albeit the orientation of the repeats is opposite to those in the *ganR* and *ganS* promoters (S4B Fig). Our results showed that neither GanR directly binds to P_*yvaB*_ (Fig 3A) nor does it regulate the expression of *yvaB* (S4C Fig). Instead, it was previously reported that another regulator YkvE negatively regulates *yvaB*, a finding that we were able to confirm (S4C Fig). Thus, our results suggest that not only is the consensus sequence of the inverted repeats important, but in addition, the orientation of the repeats is also critical for GanR binding. Again, these molecular details were not revealed in any of the previous studies.

### The consensus DNA motifs are important for GanR repression

Given the direct DNA binding of GanR and the presence of conserved DNA motifs in the promoter regions, it was plausible that GanR directly binds to any or all of the inverted repeats for repression. To confirm whether any of the DNA motifs identified through bioinformatics is important for GanR binding and to gain molecular details about GanR repression, we performed site-directed mutagenesis for those DNA motifs. To this end, point mutations altering two of the six bases were made in three of the four binding boxes within P_*ganS*_ (Boxes 1–3, Fig 3B and 3C). Modification of the final box (Box 4, Fig 3B) was avoided due to overlap of this sequence with the -35 motif of the promoter. Mutations were introduced into the wild type P_*ganS*_-*lacZ* reporter fusion constructed earlier in this study. The P_*ganS*_-*lacZ* fusions with designated point mutations in the DNA motifs (from mut1 to mut3) were similarly introduced into the wild type strain and ß-galactosidase assays were performed accordingly. Our results showed that upon modification of two bases in any of the three boxes, repression of the promoter was greatly reduced (Fig 3D).

**Fig 4:**
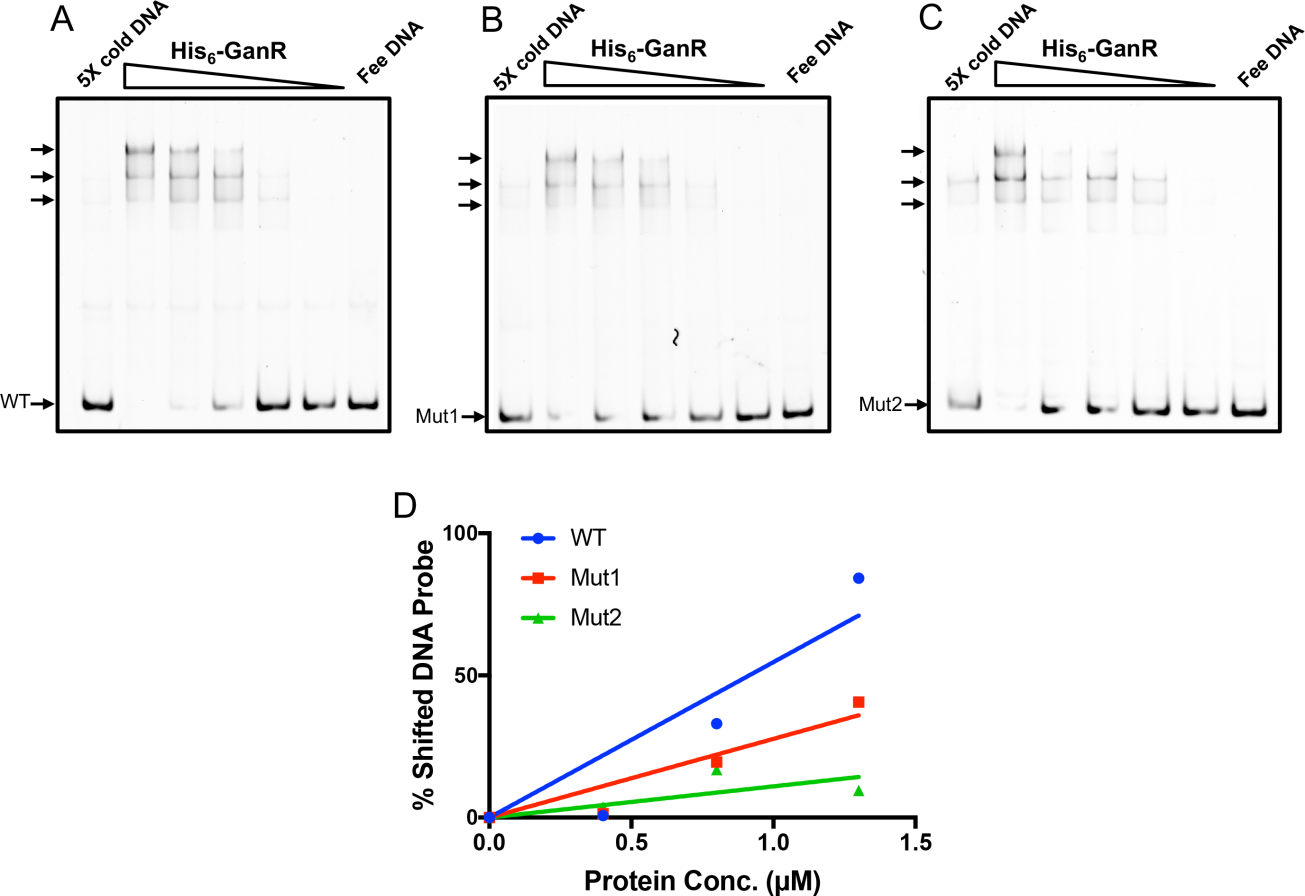
Mutations in inverted DNA repeats in P_*ganS*_ decrease GanR binding. **(A-C)** Gel mobility shift assays to determine binding of His_6_-GanR proteins to the wild type DNA sequence **(A)**, the Mut1 mutagenic sequence **(B)**, and Mut2 mutagenic sequence **(C)** of the *ganS* promoter. In all lanes, 16 ng (approximately 5 nM) fluorescent DNA probe was added. His_6_-GanR proteins were added in a range of concentrations (from 4, 1.3, 0.8, 0.4, to 0.08 μM). In each panel, the right-most lane is the fluorescent probe alone. The left-most lane contains 0.8 μM His_6_-GanR proteins, 16 ng of fluorescent probe, and 160 ng of unlabeled cold probe for competitive binding. In the upper section in each gel, shifted DNA bands were indicated by arrows. **(D)** The ratio of shifted versus total DNA was quantified from panels A-C, and graphed to show percent probe shifted versus protein concentration using WT, Mut1, and Mut2 probes.

To further support the above genetic evidence from site-directed mutagenesis and to confirm that GanR directly binds to those inverted repeats in the promoter region of *ganS*, we performed additional EMSA assays by utilizing both wild type and mutagenic P_*ganS*_ probes (Fig 3C). To complete this, we picked two mutagenic DNA probes, Mut1 and Mut2, which showed the least and the greatest depression in the above genetic study (Fig 3D), as well as the wild type *ganS* promoter. The DNA probes were PCR amplified using a primer containing 5’ labeled Cy3 dye. EMSA was performed by incubating each of the fluorescent probes with increasing amounts of GanR proteins similarly as described above. We observed a mild decrease in GanR binding when either Mut1 (Fig 4B) or Mut2 (Fig 4C) probe was used as compared to the wild type probe (Fig 4A), with the Mut2 probe showing a further decrease in binding than the Mut1 probe. The ratio of shifted versus unshifted DNAs was also calculated from the above gels. The plot of those ratios against protein concentrations for both the wild type and the two mutagenic probes was shown in Fig 4D. The results again suggested a decrease in GanR binding when mutations were introduced into the selected inverted repeats in the *ganS* promoter. In summary, our results confirmed the identified DNA motifs as the binding sequences for GanR. It also implies that strong repression likely needs cooperative binding of GanR on all four inverted repeats.

### ß-1,4-Galactobiose is an inducer of the *gan* operon

**Fig 5:**
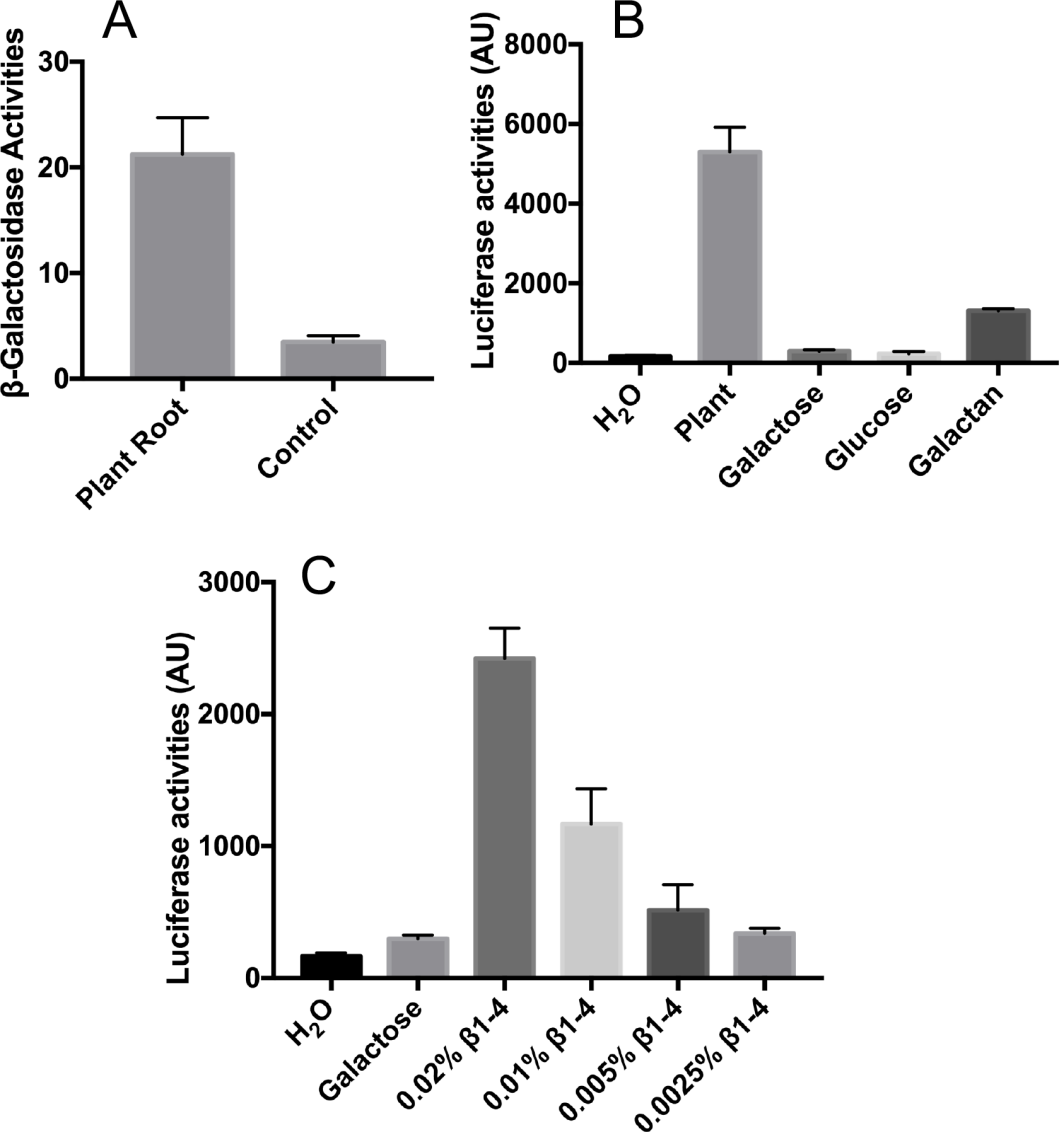
The *gan* operon can be induced by galactan, β-1,4-galactobiose, or *in situ* with plants. **(A)** *In situ* induction of the *gan* operon by using the P_*ganS*_-*lacZ* reporter strain (YC1071). Tomato plant root-associated *B*. *subtilis* reporter cells were washed off after 2 days of colonization to tomato plant roots in MG media at 25°C before assays of ß-galactosidase activities. Cells were also applied similarly to the abiotic surface (sterilized bamboo applicator sticks, Fisher Scientific) as a control. **(B)** Assays of luciferase activities from the P_*ganS*_-*lux* reporter strain (YC1146) in the presence of tomato plant root extract (5%, v/v), galactose (0.5%, w/v), glucose (0.5%, w/v), and galactan (0.05%, w/v). Cells were grown in shaking LB broth to OD_600_ = 1 and luciferase activities were measured using a plate reader (BioTek). **(C)** Similar assays of luciferase activities from the P_*ganS*_-*lux* reporter (YC1146) in the presence of galactose (0.5%, w/v), or ß-1,4-galactobiose (from 0.0025% and 0.02%, w/v). Cells were grown in LB broth with shaking at 37°C in a plate reader and bioluminescence was recorded periodically for 18 hours. The maximal fold induction of the P_*ganS*_-*lux* reporter fusion by ß-1,4-galactobiose at hour 16 in was shown here. All assays here were done multiple times and error bars represent standard deviations from those independent assays.

The *gan* operon was previously shown to be involved in utilization of galactan, a plant cell wall polysaccharide [20]. We sought to test whether this operon can be induced *in situ* when *B*. *subtilis* cells are associated with plants. We applied a *B*. *subtilis*-tomato plantlet system that we previously established to study the role of *B*. *subtilis* biofilms on root colonization [12, 14]. We then collected the P_*ganS*_-*lacZ* reporter cells (in this case without the deletion of *ganA*, YC1071) either associated with tomato plant roots or attached to an abiotic surface (see Materials and Methods), and compared the activities of those reporter cells. We observed an over 5-fold increase in the ß-galactosidase activities in root-associated cells compared to cells attached to abiotic surfaces (Fig 5A). This indicates that the *gan* operon can be induced *in situ* during bacterial colonization onto the plant roots. In addition, by using a luciferase reporter (P_*ganS*_-*lux*, we applied the luciferase-based reporter to avoid any possible feedback regulation on LacZ, see discussions below), we were also able to confirm the induction by both homogenized tomato plant root extracts (5%, v/v) and pure galactan (0.05%, w/v) (Fig 5B). Interestingly, the induction was much stronger by root extracts than pure galactan. Addition of neither glucose nor galactose caused a material induction in P_*ganS*_-*lux* in this assay (Fig 5B).

Given that GanR is a LacI-family repressor, it is possible that the allosteric inducer for GanR and for derepression of the operon is a catalytic product from galactan, rather than the polymeric galactan itself. It was previously shown that GanB hydrolyzes galactan into a mixture of galactotetraose/galactotriose/galactobiose [25]. Furthermore, in a previous study [29], it was shown that one of the hydrolyzed products of galactan, ß-1,4-galactobiose, abolishes DNA binding of GanR, indicating that ß-1,4-galactobiose could be the allosteric ligand of GanR. To further confirm the above idea by using genetic approaches, we tested potential induction of the *gan* operon by ß-1,4-galactobiose by using the luciferase reporter (P_*ganS*_-*lux*). As shown, ß-1,4-galactobiose caused a dose-dependent induction of the P_*ganS*_-*lux* reporter as indicated by luciferase activities (Fig 5C). A more than 10-fold induction was seen when ß-1,4-galactobiose was added at 0.2% (w/v), compared to the control (Fig 5C). The entire induction profile of P_*ganS*_-*lux* over a period of 18 hours in the presence of varied concentrations of ß-1,4-galactobiose was also shown in S2B Fig. We did not test induction by other types of galacto-oligosaccharides (e.g. -triose and -tetraose) since they were not commercially available. Our induction assay confirmed the biochemical result from the previous study [29].

### GanA is inhibited by its catalytic product galactose

**Fig 6:**
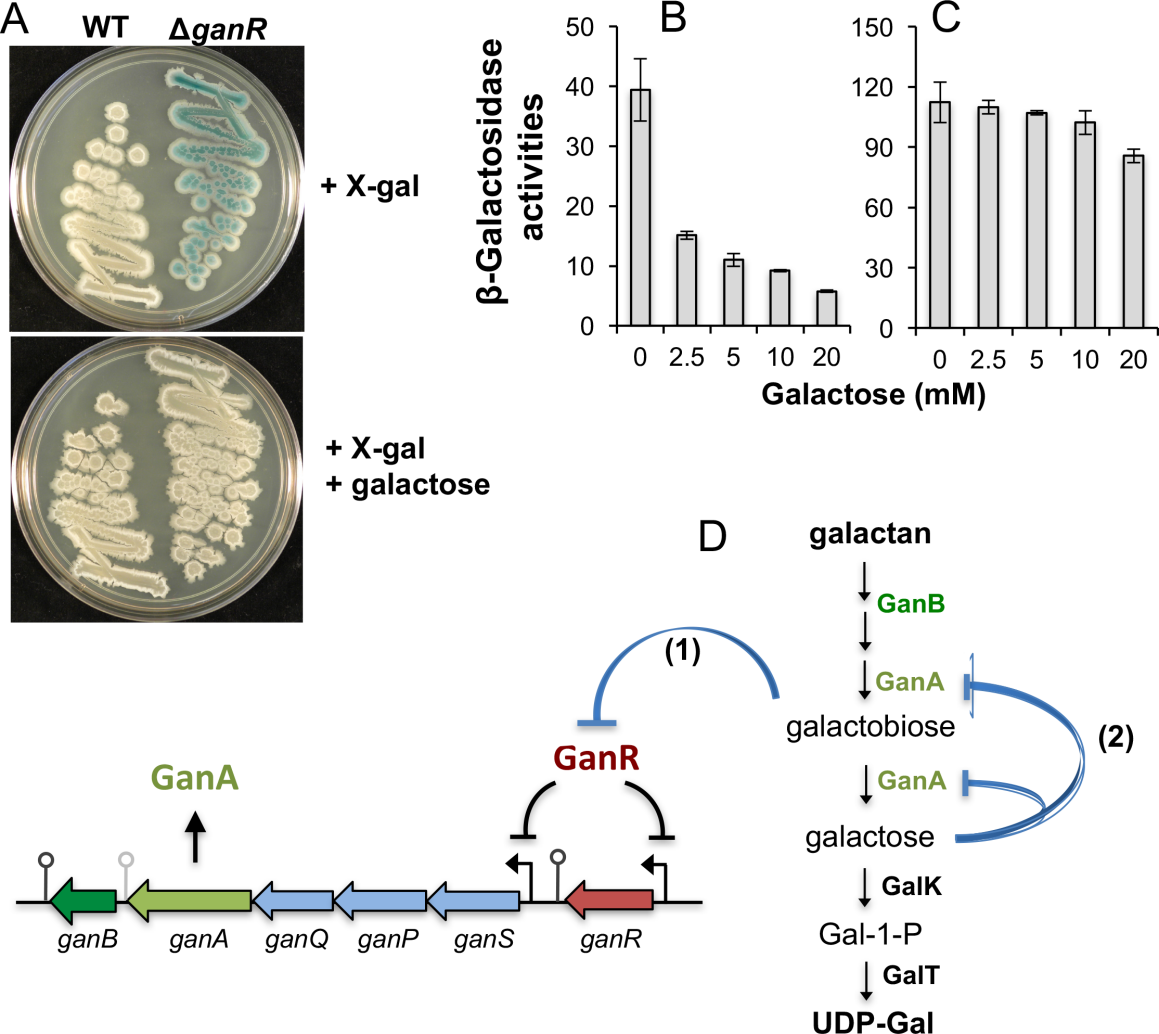
A negative feedback regulation on GanA by its catalytic product galactose. **(A)** The wild type strain (3610) and the *ganR* mutant (YC222S) were streaked out on LB plates supplemented with 40 μg ml^-1^ X-gal, and without (upper panel) or with (lower panel) galactose (0.5%, w/v). Plates were incubated at 37°C overnight before images were taken. **(B-C)** Assays of ß-galactosidase activities of protein lysates from cells expressing *ganA* (panel B, YC222S) or *lacZ* (panel C, YC1074). Assays were done in the presence of 2.5 mM ONPG and a gradient of galactose (from 2.5 to 20 mM). Error bars represent standard deviations from multiple trials. **(D)** An overview of complex regulations on galactan utilization involving both (1) a positive feedback mechanism on the transcription of the *gan* operon by ß-1,4-galactobiose and (2) a negative feedback mechanism at the protein level on GanA by its catalytic product galactose.

The *ganA* gene in the operon is known to encode a ß-galactosidase [28]. Following catabolism of galactan into galactotetraose/galactotriose/galactobiose by GanB, GanA further breaks down those oligosaccharides into galactose [25, 29]. GanA is also able to catabolize other galactose-containing glycosides such as 5-bromo-4-chloro-3-indolyl-ß-D-galactopyranoside (X-gal) and *ortho*-Nitrophenyl-ß-galactoside (ONPG). This explains why on LB plate supplemented with X-gal, the colonies of the *B*. *subtilis ganR* mutant were blue (due to overproduction of GanA and hydrolysis of X-gal by GanA; upper panel in Fig 6A). Interestingly, when galactose was added to the above plate (0.5%, w/v), it largely abolished the blue color of the colonies by the *ganR* mutant (lower panel in Fig 6A). We could think of two possible scenarios for the above result. For one, galactose inhibits *ganA* expression (but not by targeting GanR since this was observed in the *ganR* mutant). However, this possibility seems less likely since we showed earlier that addition of galactose did not significantly alter the activity of the P_*ganS*_-*lux* reporter (Fig 5B and 5C). As the other possibility, galactose acts as a competitive inhibitor for GanA.

To further test the putative inhibitory effect on GanA by galactose, we prepared cleared protein lysate from the *ganR* mutant. We then incubated the lysate with both a fixed concentration of the GanA substrate ONPG (2.5 mM) and varying concentrations of the competing galactose (from 2.5 to 20 mM). Reactions were stopped after 2 min of incubation by adding the Na_2_CO_3_ solution. The catalytic activity of GanA was assayed via ONPG hydrolysis and measurement of absorbance at 420 nm (see Materials and Methods). Our result showed that the catalytic activity of GanA (in the cleared protein lysate) was strongly inhibited by addition of galactose even when the substrate ONPG (fixed at 2.5 mM) and the putative competitive inhibitor galactose were added at the same concentration (e.g. 2.5 mM of Gal, Fig 6B). As a control, the ∆*ganR*∆*ganA* double mutant produced no activity on X-gal plate, neither in *in vtiro* ß-galactosidase assay (S5 Fig), indicating that GanA, whose gene is repressed by GanR, is the sole source of ß-galactosidase activities under our tested conditions. We next sought to compare GanA of *B*. *subtilis* to LacZ of *E*. *coli* since both of them are ß-galactosidases. We used the *B*. *subtilis* ∆*ganR*∆*ganA* double mutant bearing the P_*ganS*_-*lacZ* reporter. This strain only expresses the β-galactosidase LacZ (of *E*. *coli*) from the P_*ganS*_-*lacZ* reporter. We conducted similar assays using the cleared protein lysate prepared from that strain. Interestingly, we only observed very weak inhibition of catalytic activities of LacZ by adding same amounts of galactose (Fig 6C). Thus, even though the above assays were based on qualitative (rather than quantitative) measurements, they provide preliminary evidence that the two ß-galactosidases, GanA and LacZ, possess different feedback regulations at the protein level. Finally, in all our experiments described in this study, again, the only detectable ß-galactosidase activities were from GanA, suggesting that another ß-galactosidase enzyme YesZ, as proposed in a previous study [50], does not contribute to the overall ß-galactosidase activities under our tested conditions.

## Discussion

In this work, we have elucidated by genetic approaches the molecular regulation of the *ganSPQAB* operon, which is conserved across multiple *Bacillus* species and is involved in biofilm formation and utilization of the plant polysaccharide galactan. Our evidence reinforced the role of GanR as a transcriptional repressor of the *gan* operon and the *ganR* gene itself by directly binding to pairs of conserved DNA motifs for repression (Figs 2–4). Derepression occurs in the presence of ß-1,4-galactobiose, a partial hydrolysis product of the plant polysaccharide galactan, as well as *in situ* when cells are in association with plant roots (Fig 5). Lastly, we also showed that the ß-galactosidase GanA is strongly inhibited at the enzymatic level by galactose, the catalytic product of galactan (Fig 6). Our findings suggest a complex regulation of the galactan utilization pathway, involving both positive and negative feedback mechanisms (Fig 6D). Positive feedback regulation occurs as galactan hydrolyses to galacto-oligosaccharides (e.g. ß-1,4-galactobiose) and subsequent uptake of those oligosugars further induce expression of the *gan* operon, while breakdown from galacto-oligosaccharides to free galactose creates a rate-limiting step due to the negative feedback regulation on GanA by galactose (Fig 6D).

In addition to GanR repression, we provide evidence that the *gan* operon is also regulated by the catabolite repressor protein CcpA (Fig 2D). Catabolite repression is a conserved mechanism that allows bacteria to use preferred carbon sources and optimizes the growth rate when a complex mixture of different carbon sources are available in the environment [51]. Plant roots contain a complex and less-preferred source of carbons when compared to the more directly usable glucose and other monosaccharides. Utilization of this alternate carbon source, despite availability, casts a greater energetic requirement for the bacterial cells and may thus be considered a secondary option. The CcpA regulation on the *gan* operon could serve to provide a mechanism whereby the sensing of plant root association and utilization of carbon present therein are decoupled. Further, and more importantly, CcpA was previously shown to regulate the *galETK* genes in the Leloir pathway in *B*. *subtilis* [52, 53]. Those genes are required for further metabolism of galactose generated through galactan hydrolysis. CcpA regulation on both the *gan* operon and the *galEKT* genes thus insures simultaneous induction of all genes necessary for the complete metabolic pathway of galactan. Note that in *B*. *subtilis*, GanR does not regulate *galETK* genes (unpublished data).

Arriving at the starting point of this work, we found that plant-derived galactan can promote biofilm formation in *B*. *subtilis* (Fig 1C). In the previous study [6], we suggested that the polymeric galactan or hydrolyzed intermediate products could serve as a host signal to activate the signal transduction pathway in the bacterium, which ultimately leads to expression of genes important for matrix production and biofilm assembly (such as *epsA-O*), but it was not clear how at the time. In this study, based on predicted or demonstrated function of the proteins encoded in this operon by several recent studies [20, 25, 28, 54], we propose that the galactan utilization pathway, together with the Leloir pathway for galactose metabolism, allows *B*. *subtilis* to catabolize this common plant polysaccharide into UDP-Gal and UDP-Glu (Fig 1A). While likely being further used as carbon sources or in other biological processes, these two sugar nucleotides are essential precursors for EPS biosynthesis during *B*. *subtilis* biofilm formation (Fig 1A)[20]. We should also emphasize that the expression of *epsA-O* is not sufficient for EPS biosynthesis since sugar nucleotide precursors such as UDP-Gal and UDP-Glu are also needed in addition to the EPS assembly proteins encoded by the *epsA-O* operon. But these nucleotide sugars may not be present abundantly even when the *epsA-O* operon is induced. In addition, the biosynthetic genes for those nucleotide sugar precursors are not encoded in the *epsA-O* operon. Galactan metabolism thus provides a strategy for promoting EPS biosynthesis and biofilm formation by generating and feeding these sugar nucleotide precursors for EPS biosynthesis (Fig 1A). This strategy may become more important when *B*. *subtilis* lives in association with plant in the rhizosphere where galactan is present. In this sense, it is probably not a coincidence that the *gan* operon and the *epsA-O* operon are located almost next to each other on the *B*. *subtilis* genome with only four genes in between.

Finally, our molecular studies on GanR regulated genes and results from previous studies led us to the consensus sequence (AGTTT-4nt-AAACT) recognized by GanR (Fig 2F). We applied this consensus sequence to search for additional genes in the *B*. *subtilis* genome that may be regulated by GanR. Indeed, we identified putative GanR binding sequences in the regulatory regions in more than two dozens of genes (S6 Fig). Amongst the list is the *yukE-yueC* operon, which encodes a type VII secretion system whose function in *B*. *subtilis* is yet to be characterized [55]. In other bacteria, similar type VII secretion systems were shown to be involved in delivering virulence proteins into the host cells and therefore play an important role in bacterial pathogenesis [56]. Our bioinformatics search also identified that the promoter region of *kinB* contains multiple consensus motifs recognized by GanR. *kinB* encodes for a well-characterized sensory histidine kinase, which is involved in regulation of biofilm formation and cell differentiation by activating the master regulator Spo0A by protein phosphorylation (Fig 1A)[8]. If the presence of galactan can trigger *kinB* induction, this may provide the missing piece in our previous hypothesis (Fig 1A). In summary, further work is required in the future to determine if GanR may function as a global regulator, possibly regulating multiple pathways related to *B*. *subtilis*-plant interactions.

## Supporting information

S1 Fig Amino acid sequence alignment of GanR, MsmR, DegA, and ExuR of *B*. *subtilis*. All four proteins belong to the LacI-family transcription repressors and are predicted to regulate corresponding polysaccharide utilization gene clusters in *B*. *subtilis* (http://genolist.pasteur.fr/SubtiList/). The highly conserved arginine residues in the boxed region in the predicted DNA binding domains are highlighted in red. In the LacR^R20H^ variant, the protein lost the ability to repress the *gan* operon.
(TIFF)

S2 Fig **(A)** Assays of β-galactosidase activities of the endogenous GanA from the wild type strain(3610), the *ganR* point mutant(YC222S) and the *ganR* insertional deletion mutant(YCN217). Cells were grown in LB shaking broth to OD_600_ = 1 before harvest and analysis. Error bars represent standard deviations from three independent assays. **(B)** Induction of the *ganS* operon by ß-1,4-galactobiose. Assays of luciferase activities from the P_*ganS*_-*lux* reporter (YC1146) in the presence of galactose (0.5%, w/v), or ß-1,4-galactobiose (from 0.0025% and 0.02%, w/v). Cells were grown in LB broth with shaking at 37°C in a plate reader and bioluminescence was recorded periodically for 18 hours. All assays here were done multiple times and representative data were selected from those independent assays and shown here.
(TIFF)

S3 Fig A putative transcription attenuator in the *gan* operon. **(A) and (B)** Putative Rho-independent transcriptional terminators located in the intergenic region of *ganA* and *ganB* in *B*. *subtilis* 3610 (A) and *B*. *licheniformis* DSM14580 (B). Nucleotide sequences in green and framed from both *B*. *subtilis* and *B*. *licheniformis* resemble the putative terminator sequences. **(C)** The nucleotide sequence from *B*. *subtilis* 3610 was further analyzed and predicted to form a hairpin-like structure followed by polyU in its transcribed mRNA molecules. Free energy change (Δ*G*° = -11.2 kcal/mol) reflects relative stability of the predicted structure. Prediction of the hairpin structure and calculation of free energy were performed using RNAstructure (http://rna.urmc.rochester.edu/RNAstructureWeb/) by applying the standard analysis parameters.
(TIFF)

S4 Fig **(A)** Preparation of affinity-purified His_6_-GanR proteins. Affinity-purified proteins were size-fractionated on a 12% SDS-PAGE and were stained with Coomassie blue. The size of the protein ladder was indicated. **(B)** The putative promoter sequence of the *yvaB* gene in *B*. *subtilis*. Similar inverted DNA repeats are shown in red and underlined except that the orientation of the repeats is opposite to the consensus GanR binding sequence identified in this study (Fig 2F). **(C)** Assays of ß-galactosidase activities from the P_*yvaB*_-*lacZ* reporter in the Δ*ganA* mutant (YC1149), the Δ*ganA* Δ*ganR* double mutant (YC1150), and the Δ*ganA* Δ*ykvE* double mutant (YC1151). Cells were grown in LB shaking broth to OD_600_ = 1 before harvest and analysis. Error bars represent standard deviations.
(TIFF)

S5 Fig GanA is the sole ß-galactasidase regulated by GanR. **(A)** Wild type (3610), Δ*ganR* (YC1076), Δ*ganA* (YC1077), and Δ*ganR* Δ*ganA* double mutant (YC1078) were plated on LB plate supplemented with 40 μg ml^-1^ X-Gal. **(B)** *In vitro* ß-galactosidase assay was applied to determine ß-galactosidase activities in the indicated strains used in S5A Fig.
(TIFF)

S6 Fig A bioinformatics search for putative GanR binding sequences in the genome of *B*. *subtilis*. The search was performed by using the consensus sequence (5’-GTAAA-N4-TTTAC-3’) and the pattern search function in the Subtilist web server (http://genolist.pasteur.fr/SubtiList/) in the *B*. *subtilis* 168 genome. One mismatch in each DNA repeat was allowed and the motif search was limited to the intergenic region within 300-bp from the start codon of the candidate gene during the search. A total of about 30 genes were identified to contain putative GanR binding sequences in their promoters based on our search. The promoter of the *kinB* gene contains at least four copies of the conserved sequences.
(TIFF)

S1 Table Oligonucleotides used in this study. (PDF)
