# Supplementary figures and images for: Characterization of the regulation of a plant polysaccharide utilization operon and its role in biofilm formation in Bacillus subtilis
Source: PLoS One. 2017 Jun 15;12(6):e0179761. doi: 10.1371/journal.pone.0179761 (PMC5472308; doi:10.1371/journal.pone.0179761)

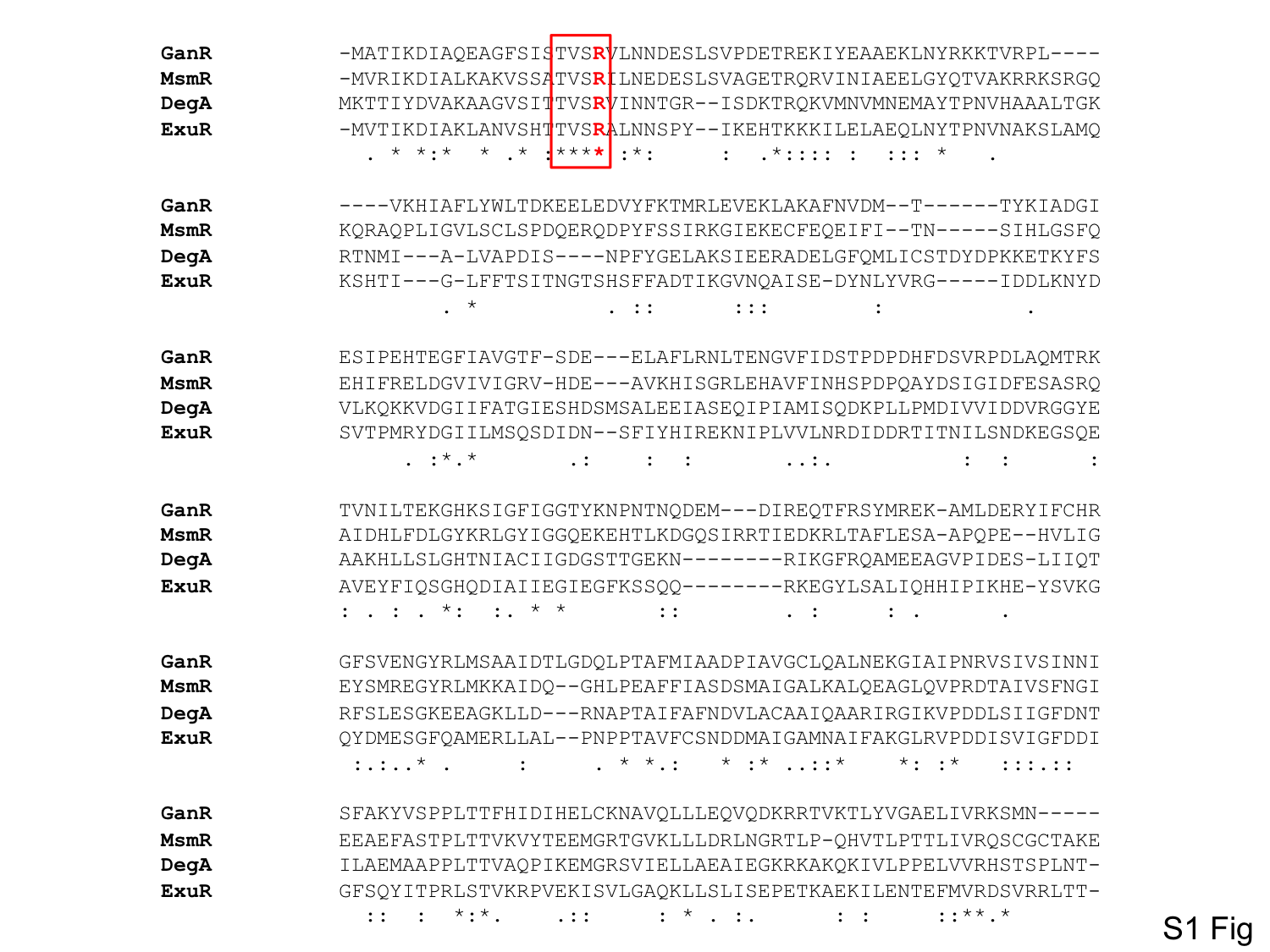

Supplement: S1 Fig — All four proteins belong to the LacI-family transcription repressors and are predicted to regulate corresponding polysaccharide utilization gene clusters in B. subtilis (http://genolist.pasteur.fr/SubtiList/). The highly conserved arginine residues in the boxed region in the predicted DNA binding domains are highlighted in red. In the LacRR20H variant, the protein lost the ability to repress the gan operon. (TIFF) [file pone.0179761.s001.tiff]

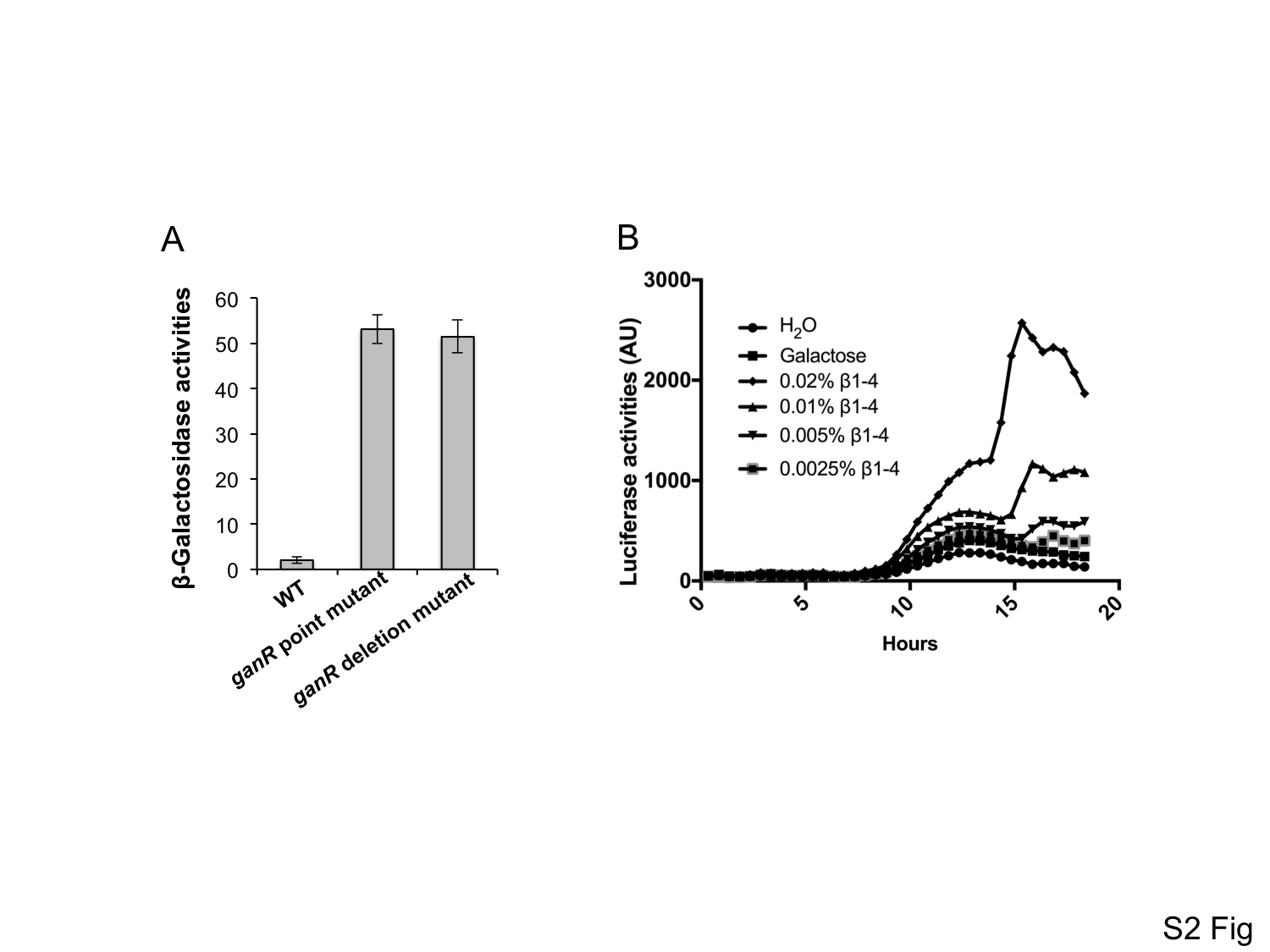

Supplement: S2 Fig — (A) Assays of β-galactosidase activities of the endogenous GanA from the wild type strain(3610), the ganR point mutant(YC222S) and the ganR insertional deletion mutant(YCN217). Cells were grown in LB shaking broth to OD600 = 1 before harvest and analysis. Error bars represent standard deviations from three independent assays. (B) Induction of the ganS operon by ß-1,4-galactobiose. Assays of luciferase activities from the PganS-lux reporter (YC1146) in the presence of galactose (0.5%, w/v), or ß-1,4-galactobiose (from 0.0025% and 0.02%, w/v). Cells were grown in LB broth with shaking at 37°C in a plate reader and bioluminescence was recorded periodically for 18 hours. All assays here were done multiple times and representative data were selected from those independent assays and shown here. (TIFF) [file pone.0179761.s002.tiff]

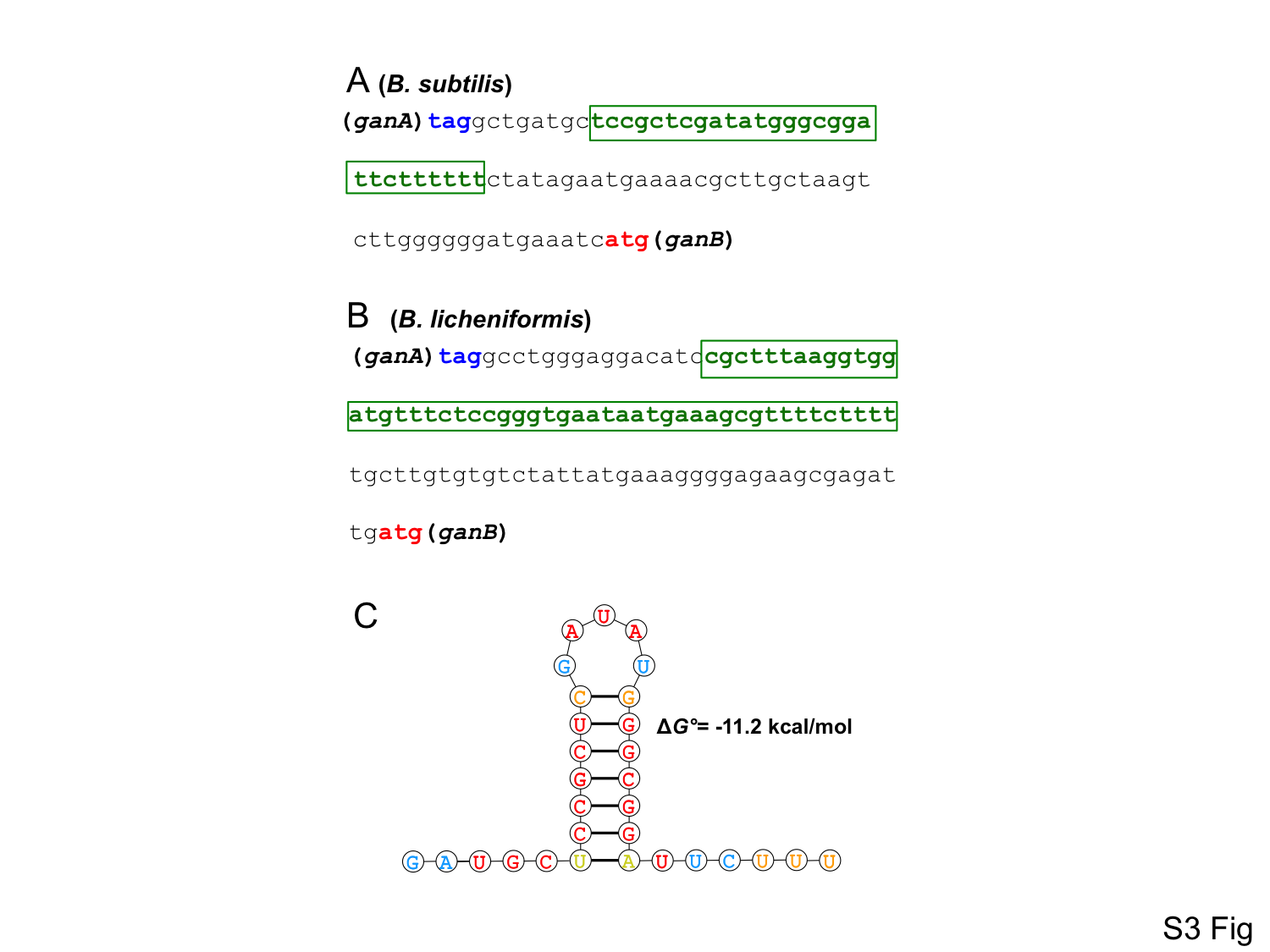

Supplement: S3 Fig — (A) and (B) Putative Rho-independent transcriptional terminators located in the intergenic region of ganA and ganB in B. subtilis 3610 (A) and B. licheniformis DSM14580 (B). Nucleotide sequences in green and framed from both B. subtilis and B. licheniformis resemble the putative terminator sequences. (C) The nucleotide sequence from B. subtilis 3610 was further analyzed and predicted to form a hairpin-like structure followed by polyU in its transcribed mRNA molecules. Free energy change (ΔG° = -11.2 kcal/mol) reflects relative stability of the predicted structure. Prediction of the hairpin structure and calculation of free energy were performed using RNAstructure (http://rna.urmc.rochester.edu/RNAstructureWeb/) by applying the standard analysis parameters. (TIFF) [file pone.0179761.s003.tiff]

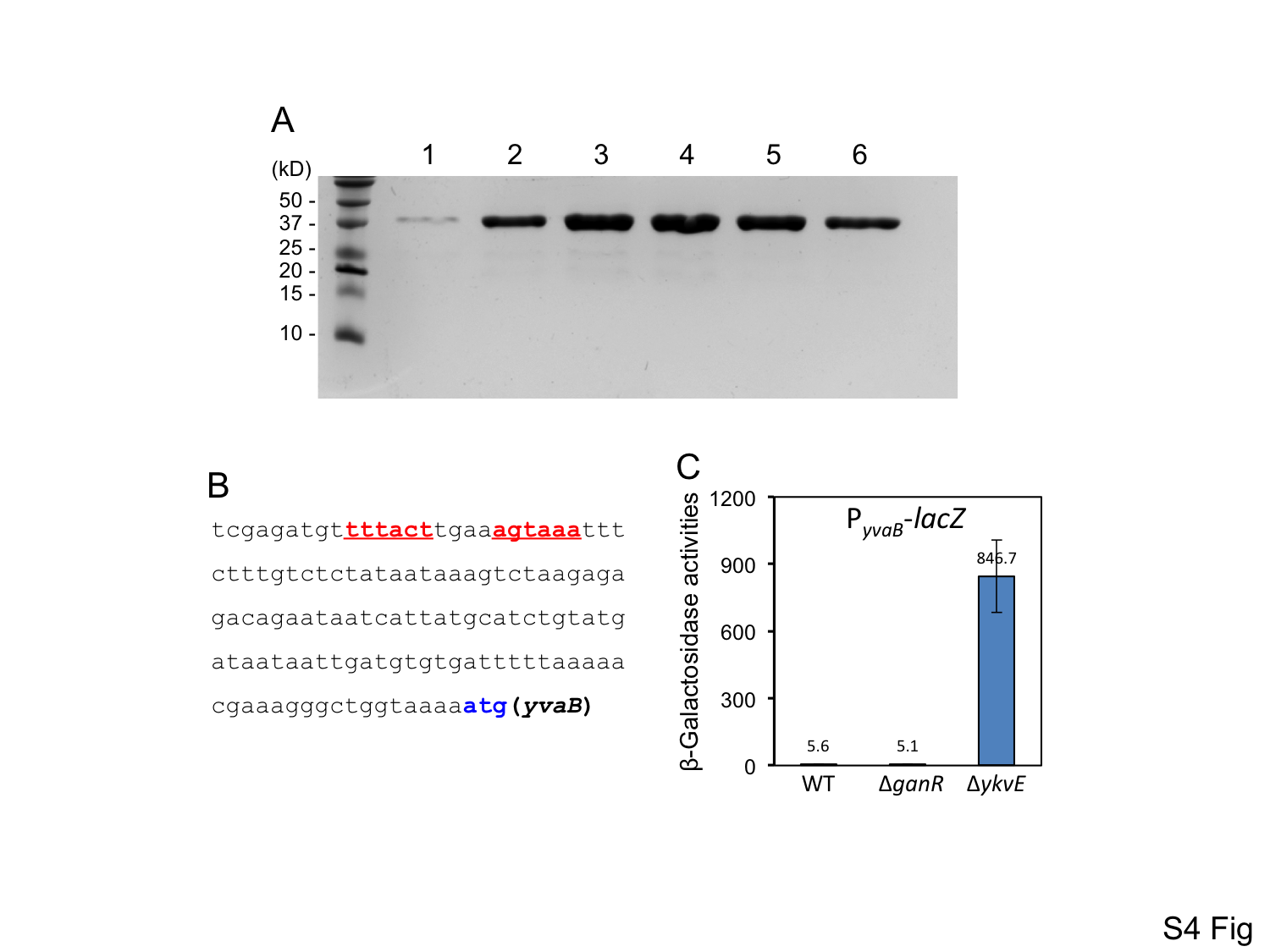

Supplement: S4 Fig — (A) Preparation of affinity-purified His6-GanR proteins. Affinity-purified proteins were size-fractionated on a 12% SDS-PAGE and were stained with Coomassie blue. The size of the protein ladder was indicated. (B) The putative promoter sequence of the yvaB gene in B. subtilis. Similar inverted DNA repeats are shown in red and underlined except that the orientation of the repeats is opposite to the consensus GanR binding sequence identified in this study (Fig 2F). (C) Assays of ß-galactosidase activities from the PyvaB-lacZ reporter in the ΔganA mutant (YC1149), the ΔganA ΔganR double mutant (YC1150), and the ΔganA ΔykvE double mutant (YC1151). Cells were grown in LB shaking broth to OD600 = 1 before harvest and analysis. Error bars represent standard deviations. (TIFF) [file pone.0179761.s004.tiff]

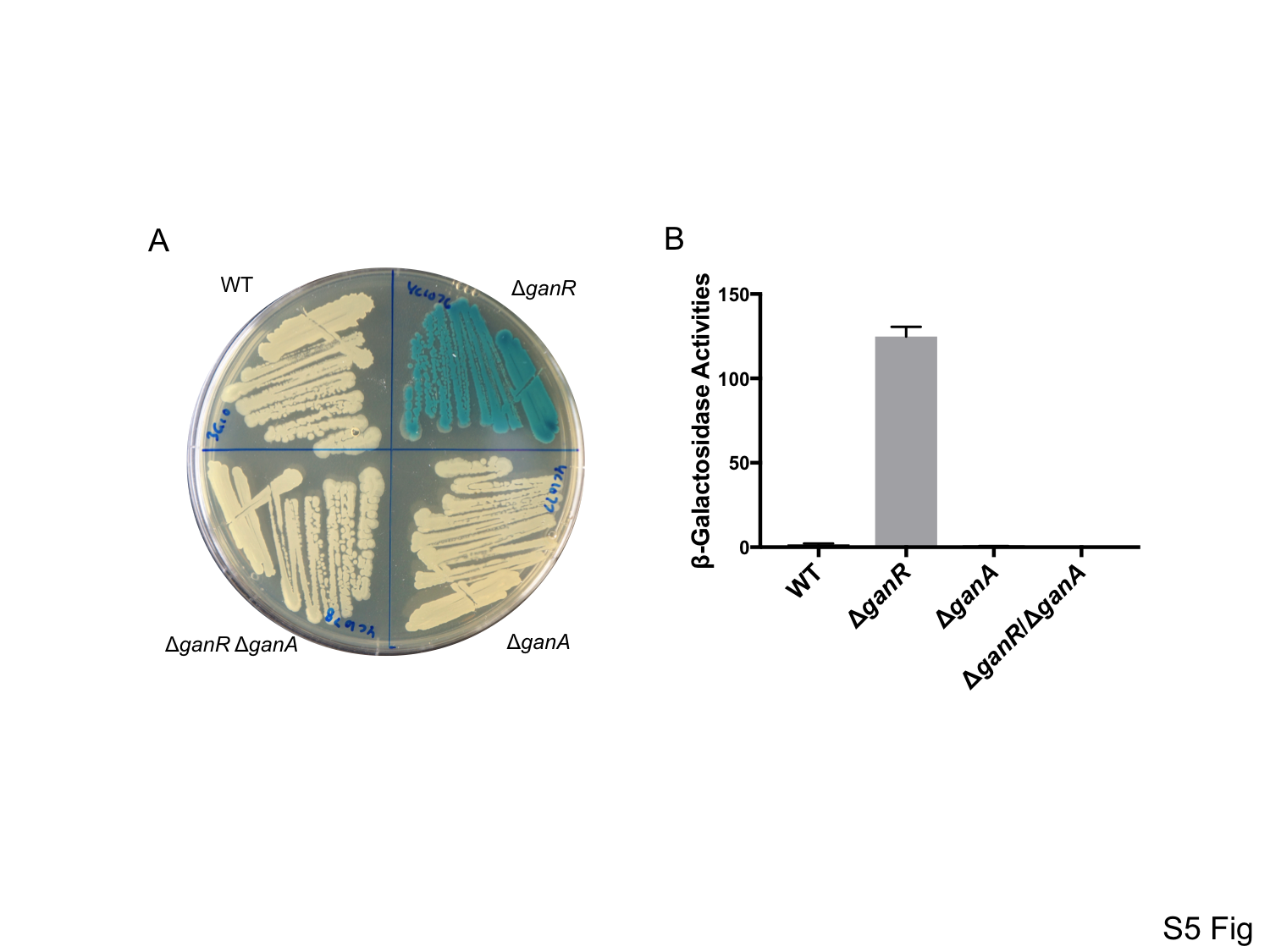

Supplement: S5 Fig — (A) Wild type (3610), ΔganR (YC1076), ΔganA (YC1077), and ΔganR ΔganA double mutant (YC1078) were plated on LB plate supplemented with 40 μg ml-1 X-Gal. (B) In vitro ß-galactosidase assay was applied to determine ß-galactosidase activities in the indicated strains used in S5A Fig. (TIFF) [file pone.0179761.s005.tiff]

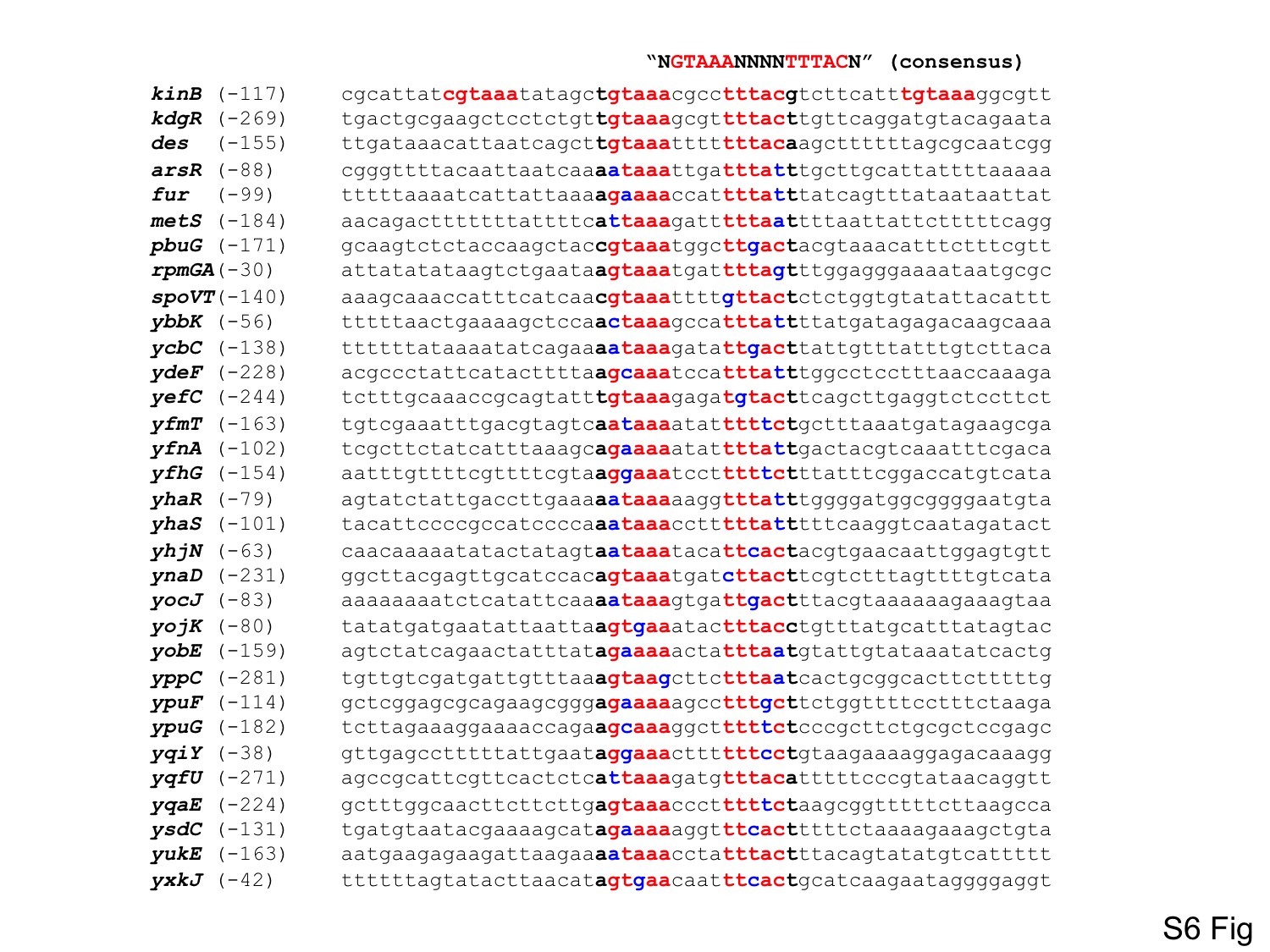

Supplement: S6 Fig — The search was performed by using the consensus sequence (5’-GTAAA-N4-TTTAC-3’) and the pattern search function in the Subtilist web server (http://genolist.pasteur.fr/SubtiList/) in the B. subtilis 168 genome. One mismatch in each DNA repeat was allowed and the motif search was limited to the intergenic region within 300-bp from the start codon of the candidate gene during the search. A total of about 30 genes were identified to contain putative GanR binding sequences in their promoters based on our search. The promoter of the kinB gene contains at least four copies of the conserved sequences. (TIFF) [file pone.0179761.s006.tiff]
