# Supplementary material for: Characterization of the regulation of a plant polysaccharide utilization operon and its role in biofilm formation in Bacillus subtilis
Source: PLoS One. 2017 Jun 15;12(6):e0179761. doi: 10.1371/journal.pone.0179761 (PMC5472308; doi:10.1371/journal.pone.0179761)
Supplement: S1 Table — (PDF) [file pone.0179761.s007.pdf]

Table S1: Oligonucleotides used in this study.

P_{ganS}-F1	5′-gtacgaattccggacccgattgcagtgggctg-3′
P_{ganS}-R1	5′-gtacggatccttcggtaggaatgaaagcgct-3′
P_{ganS}-R2	5′-gtacgcggccgcttcggtaggaatgaaagcgct-3′
P_{ganR}-F1	5′-gtacgaattccgaacggatttacaatgctgtg-3′
P_{ganR}-R1	5′-gtacgcggccgcggatccattattcagctcgcattg-3′
P_{yvaB}-F	5′-gtacgaattcccgcgctgtattccggcttgag-3′
P_{yvaB}-R	5′-gtacggatccatcacacatcaattattatcat-3′
P_{ganS}-M1F	5′-ctaagtcatttttatttagg**ct**aaaaaatttactctatgaag-3′
P_{ganS}-M1R	5′-cttcatagagtaaattttttt**ag**cctaaataaaaaatgacttag-3′
P_{ganS}-M2F	5′-tttttatttaggtaaaaaaat**gg**actctatgaagtaaatagttt-3′
P_{ganS}-M2R	5′-aaactatttacttcatagagt**cc**atttttttacctaaataaaaa-3′
P_{ganS}-M3F	5′-aaaaaatttactctatgaagt**cc**atagtttgtttacacattttc-3′
P_{ganS}-M3R	5′-gaaaatgtgtaaacaaactat**gg**acttcatagagtaaatttttt-3′
ganR-F1	5′-gtaccatatggcgacaattaaagatatcg-3′
ganR-R1	5′-gtacgaattcttaattcatactcttcctgac-3′
